# Mimicking Bone Extracellular Matrix: From BMP‐2‐Derived Sequences to Osteogenic‐Multifunctional Coatings

**DOI:** 10.1002/adhm.202201339

**Published:** 2022-08-15

**Authors:** Lluís Oliver‐Cervelló, Helena Martin‐Gómez, Nandin Mandakhbayar, Young‐Woo Jo, Elisabetta Ada Cavalcanti‐Adam, Hae‐Won Kim, Maria‐Pau Ginebra, Jung‐Hwan Lee, Carlos Mas‐Moruno

**Affiliations:** ^1^ Biomaterials Biomechanics and Tissue Engineering Group Department of Materials Science and Engineering Universitat Politècnica de Catalunya (UPC) Barcelona 08019 Spain; ^2^ Barcelona Research Center in Multiscale Science and Engineering UPC Barcelona 08019 Spain; ^3^ Institute of Tissue Regeneration Engineering (ITREN) Dankook University Cheonan 330‐714 Republic of Korea; ^4^ Department of Nanobiomedical Science & BK21 PLUS NBM Global Research Center for Regenerative Medicine Dankook University Cheonan 330‐714 Republic of Korea; ^5^ Department of Biomaterials Science School of Dentistry Dankook University Cheonan 330‐714 Republic of Korea; ^6^ Neobiotech Co. Ltd R&D Center Seoul 08381 Republic of Korea; ^7^ Department of Cellular Biophysics Growth Factor Mechanobiology group Max Planck Institute for Medical Research Jahnstraße 29 69120 Heidelberg Germany; ^8^ Institute for Bioengineering of Catalonia Barcelona 08028 Spain

**Keywords:** biomimetic peptides, cell adhesions, multifunctionality, osteogenic differentiation, RGD‐DWIVA, titanium biofunctionalization

## Abstract

Cell–material interactions are regulated by mimicking bone extracellular matrix on the surface of biomaterials. In this regard, reproducing the extracellular conditions that promote integrin and growth factor (GF) signaling is a major goal to trigger bone regeneration. Thus, the use of synthetic osteogenic domains derived from bone morphogenetic protein 2 (BMP‐2) is gaining increasing attention, as this strategy is devoid of the clinical risks associated with this molecule. In this work, the wrist and knuckle epitopes of BMP‐2 are screened to identify peptides with potential osteogenic properties. The most active sequences (the DWIVA motif and its cyclic version) are combined with the cell adhesive RGD peptide (linear and cyclic variants), to produce tailor‐made biomimetic peptides presenting the bioactive cues in a chemically and geometrically defined manner. Such multifunctional peptides are next used to functionalize titanium surfaces. Biological characterization with mesenchymal stem cells demonstrates the ability of the biointerfaces to synergistically enhance cell adhesion and osteogenic differentiation. Furthermore, in vivo studies in rat calvarial defects prove the capacity of the biomimetic coatings to improve new bone formation and reduce fibrous tissue thickness. These results highlight the potential of mimicking integrin‐GF signaling with synthetic peptides, without the need for exogenous GFs.

## Graphical abstract

Reproducing bone extracellular matrix is paramount to improve cell‐material interactions. Here, after screening bone morphogenetic protein 2‐derived sequences, biomimetic peptides with adhesive and osteoinductive capacity with a well‐defined geometrical disposition are engineered. Functionalized titanium with the peptides demonstrate to synergistically enhance mesenchymal stem cells adhesion and osteogenic differentiation and to promote new bone formation in vivo.

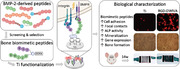

## 1 Introduction

Growth factors (GFs) are signaling molecules that play crucial roles in cell fate. Highly abundant in the extracellular matrix (ECM) of most tissues, GFs regulate cell adhesion and crosstalk with adhesion structures in cells, such as integrins.^[^ ^1^ ^]^ Furthermore, they stimulate cells by activating transmembrane receptors, which generate intracellular signals that are transduced to the cell nucleus and translated into defined biological responses, including cell growth, migration, and differentiation.^[^ ^2^, ^3^, ^4^ ^]^ Among the many described families of GFs, bone morphogenetic protein 2 (BMP‐2) has been demonstrated to be pivotal in promoting osteogenesis, both in vitro and in vivo.^[^ ^5^, ^6^ ^]^ Consequently, BMP‐2 has been combined with different types of biomaterials to promote mesenchymal stem cells (MSCs) differentiation into osteoblasts.^[^ ^7^, ^8^, ^9^, ^10^ ^]^ BMP‐2 has also been approved to be used in the clinic for treating spinal fusions and nonunion bone fractures. However, due to the short half‐life of BMP‐2, its administration normally uses supraphysiological doses, which entails serious clinical risks, such as ectopic bone formation, uncontrolled inflammation, immunological reactions and, in severe cases, death.^[^ ^11^, ^12^, ^13^, ^14^, ^15^ ^]^ Thus, GF‐based therapies, though clinically relevant for bone regeneration, call for caution and remain controversial.

In addition, promoting integrin adhesion on implant surfaces is important to enhance tissue regeneration. In this regard, biomaterial‐based strategies intended to repair and replace damaged bone tissue also rely on reproducing integrin signaling, by means of engineering surfaces with the integrin‐binding RGD motif.^[^ ^16^, ^17^, ^18^ ^]^ Although these strategies effectively improve cell attachment, proliferation, and even differentiation in vitro, they generally fail to recreate the complexity of bone ECM, with generally modest translation into animal settings.^[^ ^19^, ^20^ ^]^ Thus, to better mimic the healing microenvironment of bone, the combination of biomolecules that simultaneously trigger integrin and GF receptor signaling has been proposed.^[^ ^21^, ^22^ ^]^ In this regard, the capacity of BMP‐2 receptors to crosstalk with integrins, leading to synergistic effects in osteogenic differentiation has been illustrated in a number of studies.^[^ ^23^, ^24^, ^25^ ^]^ For instance, functionalization of hyaluronic acid hydrogels with the fibronectin (FN) type III9‐10 domain, loaded with recombinant human (rh)BMP‐2 was shown to improve osteoinduction in vivo, when compared to the delivery of the GF in nonfunctionalized hydrogels.^[^ ^26^ ^]^ Moreover, PEG hydrogels functionalized with an *α*2*β*1 integrin‐specific peptide were used as BMP‐2 delivery systems, enhancing in vivo bone formation in comparison to the delivery of BMP‐2 from collagen sponges.^[^ ^27^ ^]^ Of note, it has been recently shown that the combination of BMP‐2 with integrin binding ligands leads to synergistic osteogenic signaling with reduced doses of GF.^[^ ^23^, ^27^, ^28^, ^29^ ^]^

Nonetheless, in order to avoid the aforementioned risks of rhBMP‐2 in clinical settings, the development of synthetic peptides recapitulating the osteogenic domains of BMP‐2 is gaining increasing attention. These synthetic peptides can be immobilized on the biomaterial surface at high densities, providing local and targeted effects, without being released into the bloodstream and thus avoiding unwanted off‐target effects. Peptides are structurally simpler, more stable than proteins, and cheaper to produce. They also display higher specificity and are less likely to induce an immune response.^[^ ^30^, ^31^ ^]^ In particular, a peptidic fragment derived from the knuckle epitope of BMP‐2 (spanning residues 73–92 – KIPKASSVPTELSAISTLYL), which binds to BMP receptor type II,^[^ ^32^ ^]^ promotes osteodifferentiation.^[^ ^33^ ^]^ On the other hand, the wrist epitope (amino acids 30–34 – DWIVA), which primarily interacts with BMP receptor type I,^[^ ^34^ ^]^ has also been identified and demonstrated to display osteogenic potential.^[^ ^35^ ^]^ Based on the rationale discussed above, these BMP‐2 domains may be integrated with RGD‐based ligands to induce integrin/GF signaling without the need of using GFs.^[^ ^36^ ^]^ Accordingly, the functionalization of different substrates with the KIPKASSVPTELSAISTLYL and RGD peptides proved to synergistically promote osteogenic differentiation and mineralization, although the potential of this strategy in vivo was not investigated.^[^ ^37^, ^38^ ^]^ Similarly, tethering both peptides onto alginate hydrogels improved MSCs differentiation toward the osteogenic lineage.^[^ ^39^ ^]^ Interestingly, in the same work, the combination of RGD and the wrist‐derived DWIVA motif did not have such a positive effect.

Based on these evidences and trying to elucidate the capacity of BMP‐2‐derived peptides to promote osteogenesis, herein, we report on a small library of synthetic peptides derived from the wrist and knuckle epitopes of BMP‐2. The osteogenic potential of such peptides was screened with C2C12 cells and the most bioactive sequences were subsequently combined with integrin‐binding RGD motifs (linear and cyclic) within an engineered biomimetic peptide, aiming at mimicking bone ECM and exploiting integrin and GF signaling. Thus, multifunctional peptides with a spatially defined geometry of the bioactive sequences were synthesized and further used to functionalize titanium surfaces. Physiochemical characterization of the materials demonstrated the successful surface coating. The feasibility of this strategy was then corroborated by means of cell adhesion and osteodifferentiation assays with human MSCs. The biomimetic peptides were able to enhance cell adhesion and osteogenic differentiation in comparison to controls. Moreover, such biomimetic approach was validated in vivo in a model of calvarial defect in rats, promoting an improvement of new bone growth and reduced formation of fibrous tissue on functionalized titanium implants. Thus, we demonstrate for the first time the potential of using multifunctional biomimetic peptides promoting integrin‐GF receptor local interactions to mimic bone ECM microenvironment and to trigger osteoinduction and bone formation on biomaterials that are clinically relevant, such as titanium.

## 2 Results and Discussion

### 2.1 Design of BMP‐2‐Derived Peptides with Osteogenic Potential

BMP‐2 is a GF that belongs to the transforming growth factor beta (TGF‐*β*) superfamily, which has been demonstrated to be pivotal in osteogenesis.^[^ ^5^ ^]^ In particular, BMP‐2 has two binding epitopes (wrist and knuckle) to interact either with type I or type II transmembrane serine/threonine kinase receptors (BMPR‐I and BMPR‐II, respectively). Such interactions trigger Smad dependent or independent signaling, activating in both cases osteogenic genes.^[^ ^40^, ^41^, ^42^ ^]^ Therefore, these two epitopes represent ideal regions of the protein to screen for synthetic peptides, aiming at developing peptidic sequences with osteogenic potential but devoid of the inherent clinical risks described for the use of the entire GF.

The wrist epitope of BMP‐2 is assembled around the central *α*‐helix (*α*3) of the protein and binds with high affinity to BMPR‐I. It is also large and discontinuous and it presents elements of the two BMP‐2 monomers. On the contrary, the knuckle epitope mainly interacts with the BMPR‐II (although with low affinity) and it comprises binding residues from only one monomer, which are located in different *β* sheets, mainly in *β*3, *β*4, *β*6, and *β*7.^[^ ^43^ ^]^ Interestingly, most of the key binding residues of the wrist epitope of BMP‐2 are invariant or replaced by isofunctional side chains in BMP‐7, suggesting that only a small subset of residues is essential to determine binding specificity.^[^ ^44^ ^]^

**Figure 1:**
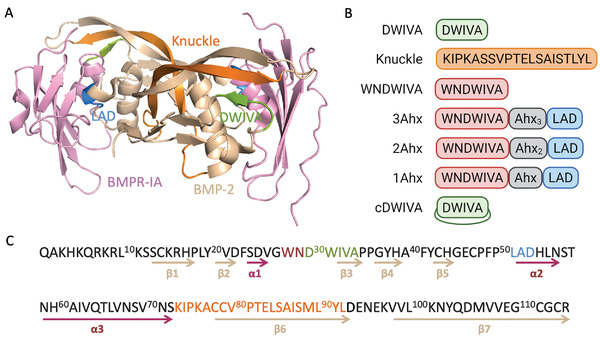
A) Ribbon representation of the BMP‐2‐BMPR‐IA complex (PDB, code 1REW). Residues 30–34 corresponding to the DWIVA sequence are highlighted in green. Knuckle sequence (residues 73–92) is highlighted in orange and the LAD motif (residues 51–53) in blue. The two monomers of BMP‐2 are shown in light brown and the BMPR‐IA in light pink. B) Schematic representation and nomenclature of the designed peptides covering BMPRs‐binding domains. C) BMP‐2 sequence with the corresponding primary and secondary structure, in which *α*‐helixes are depicted in maroon and *β*‐strands in light brown (based on PDB, code 1REW).

Based on the crystal structure of BMP‐2 and its interaction with the BMPR‐I and BMPR‐II, it has been possible to identify domains that play a crucial role in the osteodifferentiation process. The first peptide derived from BMP‐2 and combined with biomaterials found in the literature was obtained from the 73–92 residues of the knuckle epitope, belonging to the *β*6‐strand of BMP‐2. From the original sequence, the cysteine residues 78 and 79 were mutated to serine (C78S, C79S) and methionine was replaced by threonine (M89T), resulting in the KIPKASSVPTELSAISTLYL motif (**Figure** **1**).^[^ ^45^ ^]^ Such peptide should mainly interact with BMPR‐II, although with low affinity.^[^ ^43^ ^]^ However, it was also shown that this peptide antagonized BMP‐2 binding to both type I and type II BMP receptors, promoted an overexpression of alkaline phosphatase (ALP) activity and osteocalcin and also induced ectopic bone formation in rat calf muscle within 3 weeks. As such, the KIPKASSVPTELSAISTLYL motif based on the knuckle sequence is considered to be able to promote osteogenic differentiation.^[^ ^45^ ^]^

In subsequent studies, an alternative peptide derived from the wrist epitope was also found to be osteoinductive. In particular, the DWIVA sequence (residues 30–34 of BMP‐2, Figure 1) was demonstrated to enhance mineralization and induce the overexpression of pSMAD protein and ALP activity of MSCs. It was also observed that DWIVA interacts mainly with BMPR‐IA.^[^ ^35^ ^]^ These results are in accordance with a previous report by Kirsch et al., in which it was found that residues D30 and W31, located in the turn previous to the *β*3‐strand, had a high affinity toward BMPR‐IA, while the A34, located in the *β*3‐strand, had more affinity toward BMPR‐II, although the extent of this interaction was lower.^[^ ^43^ ^]^

On the basis of these seminal studies, both the knuckle and wrist‐derived peptides have been widely used alone or in combination with biomaterials to induce osteogenic differentiation.^[^ ^33^, ^39^, ^45^, ^46^ ^]^ Nonetheless, the osteogenic capacity of the wrist epitope remained controversial, as illustrated by a work from Madl et al. in which the DWIVA peptide was shown to lack osteoinductive potential.^[^ ^39^ ^]^

In addition to the knuckle and wrist regions, the structural analysis of BMP‐2 has also revealed the importance of the residues L51 and D53 of BMP‐2, both acting as a hotspot for the binding of BMPR‐IA. Indeed, mutation of L51 to P51 decreased the binding affinity toward BMPR‐IA.^[^ ^47^, ^48^ ^]^ Moreover, the substitution of alanine 52 by arginine (A52R) increased the dissociation rate of BMPR‐IA.^[^ ^43^ ^]^ Thus, the LAD motif may also be relevant in the BMP‐2‐BMPR‐IA interaction. This motif is located in the *α*2‐helix of BMP‐2 and it should be remarked that, although both DWIVA and LAD motifs interact with high affinity with BMPR‐IA, each one binds to a different monomer of the receptor.^[^ ^44^ ^]^

Taking into consideration, these findings and based on the crystal structure of the interaction between BMP‐2 and BMPR‐IA, we decided to design a small library of synthetic peptides covering mainly BMPR‐IA‐binding domains within BMP‐2 in order to identify bioactive sequences with osteogenic potential (Figure 1). Both, the KIPKASSVPTELSAISTLYL sequence (herein named “knuckle peptide”) and the DWIVA peptide (derived from the wrist region) were originally included in the library. However, the DWIVA motif, which is a much shorter peptide, was further modified to engineer alternative wrist‐derived peptides. One such analogue was designed by incorporating at the N‐terminus of the DWIVA sequence the two preceding residues naturally occurring in BMP‐2 (W28 and N29), giving rise to the WNDWIVA sequence. The rationale behind this modification was to cover a wider range of the wrist epitope and to include the W28 residue, which is conserved in all main BMPs involved in bone regeneration, namely BMP‐4, ‐6, ‐7, and ‐9.^[^ ^49^, ^50^, ^51^ ^]^ Furthermore, to study the importance of the LAD motif in the interaction with the BMPR‐IA, we designed peptide variants fusing the WNDWIVA and LAD sequences. In detail, the two epitopes were separated by three aminohexanoic acids (3Ahx), which intended to mimic the natural space between both motifs in the native BMP‐2. Indeed, such distance in the protein is of 23.11 Å, whereas our spacer measures 25 Å, reproducing with precision the native distance. In addition, two other analogues with shorter spacers (2Ahx and 1Ahx) were used to study the influence of the spacing between both sequences. Thus, the incorporation of either three, two, or just one Ahx lead to the WNDWIVA‐Ahx_3_‐LAD, WNDWIVA‐Ahx_2_‐LAD, and WNDWIVA‐Ahx‐LAD peptides, respectively. Finally, a cyclic analogue of the DWIVA peptide (i.e., cDWIVA) was produced to study the effect of conformational restriction of the wrist motif in its biological activity.

The selected sequences were manually synthetized by means of standard solid‐phase peptide synthesis (SPPS) methods, following the Fmoc/tBu strategy and protocols optimized in our research group. Peptide synthesis details can be found in the Experimental Section, whereas their chemical structures are shown in Figure S1 (Supporting Information).

### 2.2 Screening of BMP‐2‐Derived Peptides with Osteogenic Potential

**Figure 2:**
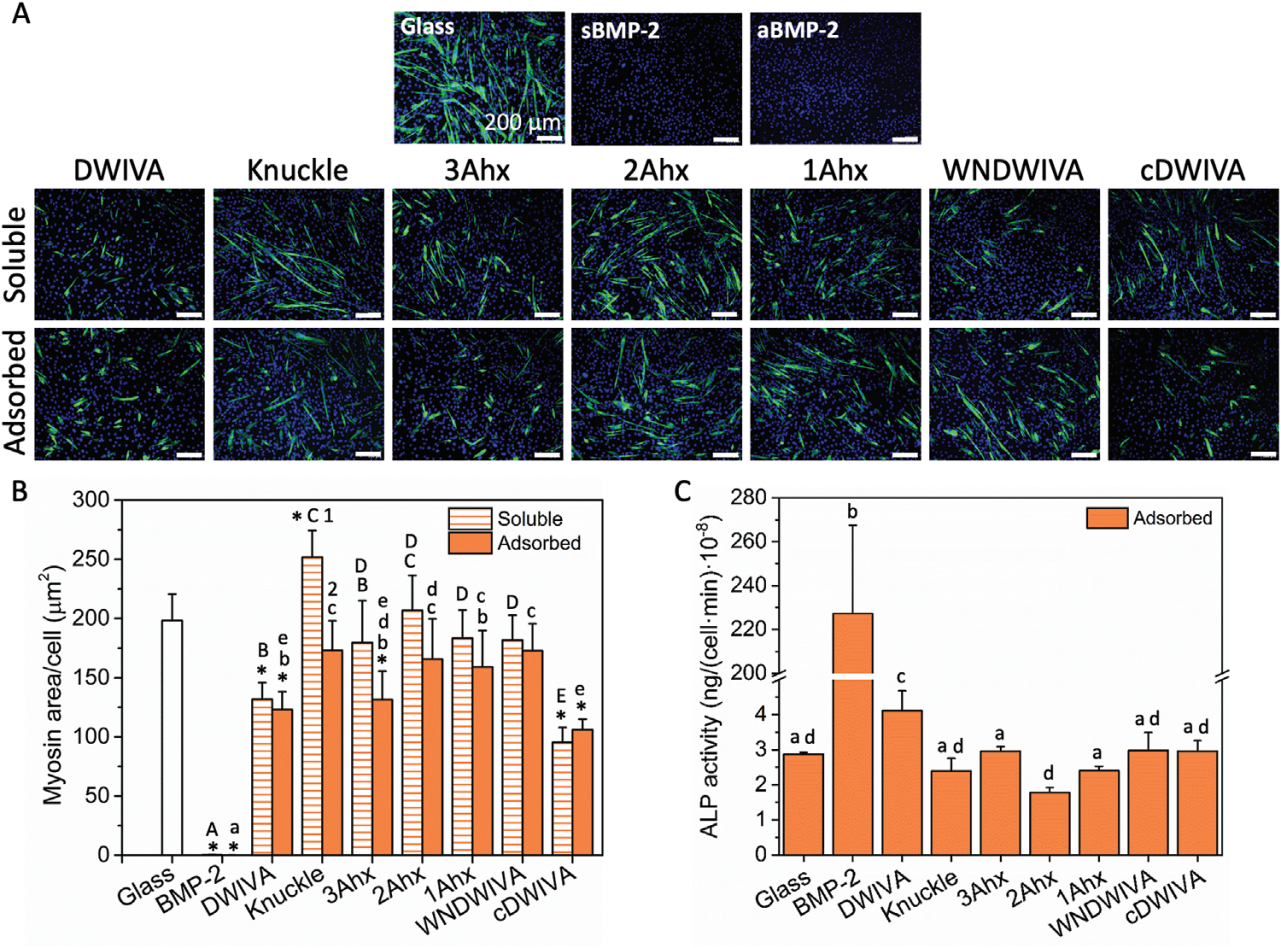
Evaluation of the osteogenic capacity of the BMP‐2‐derived peptides. A) C2C12 myosin heavy chain (MHC) staining of multinucleated myotubes after 6 days of culture on the indicated surfaces (scale bar = 200 µm). B) Myosin area quantification relative to the cell number. * represents statistically significant differences in comparison to the glass control. Different capital letters denote statistically significant differences between soluble peptides, whereas small letters denote statistically significant differences between adsorbed peptides. Different numbers denote statistically significant differences between the soluble and the adsorbed conditions for a particular peptide (*p* < 0.05). C) ALP activity after 8 days in culture of the different peptides on the adsorbed condition. Different letters denote statistically significant differences between peptides (*p* < 0.05). Each condition was replicated in triplets in each experiment (*n* = 3) and five pictures per sample were used to quantify myosin area.

To check the osteogenic potential of the synthesized peptides, a screening with C2C12 myoblasts was performed. These cells are able to fuse and form myotubes under standard culturing conditions. However, in the presence of BMP‐2, myogenic differentiation is inhibited, and C2C12 start to transdifferentiate in bone cells.^[^ ^52^, ^53^ ^]^ Thus, C2C12 myoblasts were cultured onto glass substrates and stimulated either with soluble or adsorbed peptides on the substrate. After 6 days in culture, their capacity to inhibit myogenesis was evaluated by immunostaining of myosin heavy chain (MHC) (**Figure** **2**A).

As expected, well‐formed myotubes were observed on glass control, while the presence of BMP‐2 (either adsorbed on the surface or in soluble form) totally inhibited MHC formation. Interestingly, the DWIVA and cDWIVA motifs, both in their soluble and adsorbed modes, were able to significantly inhibit myotube formation, representing the two most active peptides within the synthesized analogues (Figure 2B). In contrast, the WNDWIVA sequence, which covers a slightly larger area of the wrist region, failed to reproduce the same biological effect. The analogues incorporating the LAD sequence separated by 1 or 2 Ahx (WNDWIVA‐Ahx_2_‐LAD or WNDWIVA‐Ahx‐LAD, respectively) were also inactive. However, the adsorbed 3Ahx analogue did show a statistically significant inhibition of myotube formation, which indicates a positive effect of including the LAD sequence together with WNDWIVA, only when the distance found between the two motifs in the native BMP‐2 is preserved. In addition, most of the adsorbed peptides had a tendency to higher suppress myogenesis in comparison to the soluble ones, which stresses the importance of presenting the active moieties in the adsorbed form to drive cell response. In this regard, administration of soluble GFs has shown to be less effective in regulating cell fate in comparison to matrix‐bound GFs, where controlled and sustained influence in cell behavior is achieved with much lower doses of GF.^[^ ^52^, ^54^ ^]^

Taken together, these results confirm the capacity of the DWIVA sequence to, at least partially, mimic BMP‐2 potential in blocking myogenesis of C2C12 cells. This pentapeptide, well conserved in other BMPs involved in bone regeneration such as BMP‐4,^[^ ^55^, ^56^, ^57^ ^]^ seems to be structurally optimized in its native form, as even subtle changes result in loss of activity. This may be attributed to detrimental conformational changes introduced in the longer sequences, although the precise mechanisms should be studied with further detail. It is also worth mentioning that the knuckle epitope was inactive in our cell model. The discrepancies with previous reports^[^ ^39^, ^45^, ^58^ ^]^ may arise from differences in the cell type used, peptides concentration, and mode of presentation. Indeed, in the work of Saito et al., the knuckle peptide was physically adsorbed on polystyrene at a concentration of 3.1 µg mm^−2^, whereas the concentration in the present work was of 0.6 µg mm^−2^, which is about five times lower.^[^ ^45^ ^]^ Similarly, Madl et al. tested the osteogenic capacity of the knuckle sequence on osteoblasts in contrast to the present work, where C2C12 cells were used.^[^ ^39^ ^]^ The aforementioned differences thus do not allow for a direct comparison between the studies.

Next, the BMP‐2‐derived peptides were adsorbed on glass and evaluated toward C2C12 expression of ALP activity after 8 days in culture (Figure 2C). Interestingly, only the DWIVA peptide showed a statistically significant increase of ALP activity in comparison to the nonfunctionalized glass, which corroborated its capacity to promote the osteodifferentiation of C2C12 cells. These results are in accordance with a previous study from Lee et al., in which DWIVA had the capacity to induce ALP activity and improve mineralization.^[^ ^35^ ^]^

Thus, the DWIVA motif was selected as potential candidate for further studies in combination with the RGD sequence, aiming to engineer advanced biomimetic peptides with capacity to simultaneously promote synergistic integrin and GF signaling.

### 2.3 Development of Multifunctional Biomimetic Peptides

**Figure 3:**
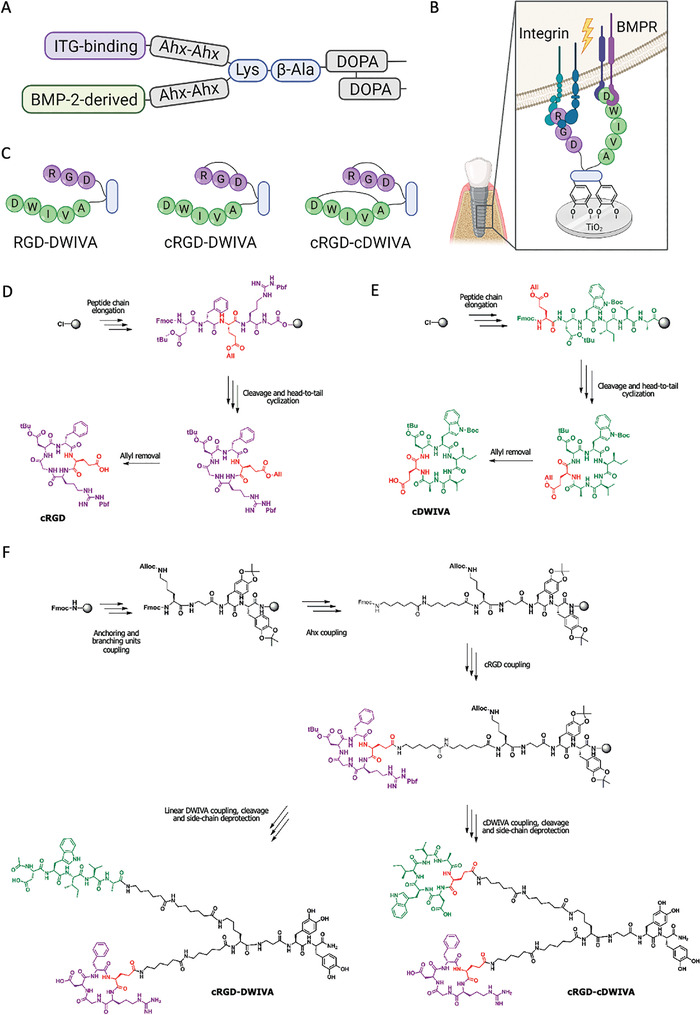
A) Schematic representation of the main elements of the biomimetic peptides. B) Integrin‐GF receptor crosstalk activated by the biomimetic peptide. C) Schematic representation of the biomimetic peptides. Created with BioRender.com. D) Chemical synthesis of the cRGD. E) Chemical synthesis of the cDWIVA. F) Chemical synthesis of the cRGD‐DWIVA and cRGD‐cDWIVA biomimetic peptides.

The combination of the DWIVA motif with the integrin‐binding peptide RGD was accomplished by incorporating the two sequences in a synthetic biomimetic peptide, in which the spatial orientation of both sequences is chemically defined (**Figure** **3**A). The copresentation of the bioactive sequences with a defined spatial control proved crucial to effectively engage in integrin‐GF crosstalk at the ventral side of the cell in a previous study^[^ ^59^ ^]^ (Figure 3B).

Thus, in order to develop ECM‐biomimetic peptides, three tailor‐made synthetic peptides were envisioned: i) the RGD‐DWIVA peptide; ii) a variant in which the RGD sequence was presented in its cyclic version (i.e., cRGD‐DWIVA); and iii) a third molecule containing both motifs in their cyclic conformation (i.e., cRGD‐cDWIVA) (Figure 3C). The rationale for using cyclic RGD was based on the well‐established effect that conformational restriction has on the integrin binding capacity of the peptide. Indeed, cyclization of linear RGD into cyclo(RGDfX) significantly improves its activity and selectivity toward *α*v*β*3 integrins, which are highly expressed in bone forming cells, and in particular, in MSCs.^[^ ^60^, ^61^, ^62^, ^63^ ^]^ In contrast, the role of cyclization of the DWIVA sequence in its BMPR‐binding activity remains unknown. In our hands, cDWIVA did not improve ALP activity in C2C12 cells (Figure 2C), however, it did suppress myogenesis (Figure 2B), and thus was also considered to study the influence of such conformational constraint in the biological performance of the biomimetic peptide.

The synthetic protocol to produce RGD‐DWIVA has been previously optimized^[^ ^59^ ^]^ and is based on the solid‐phase assembly on Fmoc–Rink Amide resin using SPPS methods. The synthesis started with the addition of two units of l‐3,4‐dihydroxyphenylalanine (DOPA), which act as anchoring groups to bind metallic oxide surfaces. In detail, the catechol groups interact with metallic surfaces by coordinative interactions.^[^ ^64^, ^65^, ^66^, ^67^, ^68^ ^]^ Such binding has been described to be very stable under wet conditions and has the advantage of providing a simple one‐step functionalization method, considerably reducing the number of reaction steps compared to other conventional processes, such as silanization.^[^ ^69^, ^70^ ^]^ Subsequently, a beta‐alanine and an orthogonally protected lysine (Fmoc/Alloc) were coupled to the resin. Lysine is a key residue in the synthetic strategy as it serves as branching point in the peptide and allows the sequential and selective assembly of the two bioactive sequences. Finally, each branch of the dimeric architecture contained two units of Ahx, designed as linkers to provide the adequate spacing of the RGD and DWIVA motifs, a prerequisite to ensure optimal signaling.^[^ ^59^ ^]^

The synthesis of the two other analogues (i.e., cRGD‐DWIVA and cRGD‐cDWIVA), however, was more challenging, as it required the incorporation of cyclic sequences into the biomimetic peptide. In this case, taking into account that cyclization in solid‐phase was problematic, the peptides were cyclized first in solution and then coupled to the resin‐bound peptide in solid‐phase (Figure 3D,E,F). To this end, the linear peptides were synthesized in 2‐chlorotrityl chloride, cleaved with a low percentage of TFA and cyclized head‐to‐tail in solution. Moreover, the cyclic peptides were prepared using an orthogonal strategy and incorporated a glutamic acid protected with an allyl group. Such design allowed the selective removal of the allyl function using palladium chemistry, yielding the cyclic peptides with a free carboxylic function without affecting the other protecting groups. The complete details of the synthesis as well as the characterization of the peptides is described in the Experimental Section.

### 2.4 Biomimetic Peptides are Immobilized on Ti Substrates

**Figure 4:**
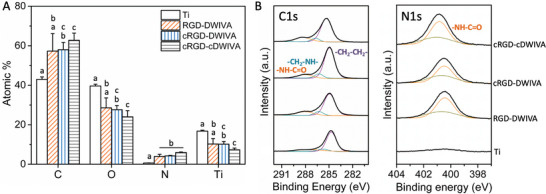
Physicochemical characterization of Ti substrates functionalized with either RGD‐DWIVA, cRGD‐DWIVA, or cRGD‐cDWIVA peptides. A) Atomic percentages (%) of C1s, O1s, N1s, and Ti2p measured by XPS. B) High resolution spectra of deconvoluted C1s (left) and N1s (right). Duplicates per condition were used for XPS measurements. Different letters denote statistically significant differences between groups (*p* < 0.05).

Immobilization of the biomimetic peptides (RGD‐DWIVA, cRGD‐DWIVA, or cRGD‐cDWIVA) on Ti surfaces was performed by a simple one‐step procedure adding one drop of a 100 µm solution of the peptidic biomolecule on top of the disks and incubated overnight, taking advantage of the high affinity of catechol groups to Ti oxide.^[^ ^71^, ^72^ ^]^ Afterward, physicochemical characterization of the functionalized surfaces was performed by means of XPS, contact angle, Raman spectroscopy, and fluorescent measurements (**Figure** **4**; and Figure S2, Supporting Information).

Surface chemical composition of the functionalized Ti substrates was evaluated by XPS measurements. As represented in Figure 4A, the uncoated Ti disks presented the highest concentrations of oxygen (O 1s) and titanium (Ti 2p), associated to the Ti oxide layer. However, the nitrogen concentration (N 1s), which is commonly attributed to the amide bonds of the peptidic molecules, was almost undetected. In this regard, the nitrogen signal significantly increased up to ≈4% upon functionalization with the biomimetic peptides, reaching statistically comparable levels for the three conditions. The presence of the peptides was additionally confirmed by a concomitant decrease in the concentrations of oxygen and titanium and an increase in the carbon content, which is an indicator that the adherent peptides mask the TiO_2_ signal. Indeed, such modifications in the surface chemistry have been associated to a deposition of a peptide layer on top of Ti surfaces in previous works.^[^ ^73^, ^74^, ^75^ ^]^ Deconvolution of high resolution C1s spectra of control Ti revealed a main peak at 285.2 eV (purple color in Figure 4B), which is associated to aliphatic —CH_2_—CH_2_— bonds of organic contaminants. In contrast, when Ti was functionalized with the biomimetic peptides, characteristic peaks at high binding energy, corresponding to —C—N/—C—O bonds (286.7 eV) and amide groups (288.4 eV), were also observed (blue and orange colors in Figure 4B), in agreement with the literature.^[^ ^76^, ^77^ ^]^ Moreover, the presence of the molecules was also confirmed by the high resolution spectra of N1s, in which characteristic peaks of amide groups at a binding energy of 400.5 eV were also detected.

To further demonstrate the successful functionalization of Ti substrates, contact angle measurements were performed (Figure S2A, Supporting Information). Taking into account that the three biomimetic peptides share the same chemical composition, these studies were only done with the RGD‐DWIVA condition. Ti functionalization with the RGD‐DWIVA molecule resulted in a significant increase of its surface wettability compared to nonfunctionalized substrates, as shown by a decrease in the water static contact angle. Such increase in hydrophilicity was expected due to the presence of charged and polar amino acids in the biomolecule and is well in accordance with previous studies using this type of molecules.^[^ ^59^, ^75^ ^]^ In addition, quantification of the density of peptide bound on the surface was calculated by using a fluorescently labeled RGD‐DWIVA model peptide (F‐RGD‐DWIVA), according to a protocol established in our lab. In short, the fluorescent peptide was detached from the surfaces by using basic etching under 70 °C, and the obtained fluorescence was correlated to a peptide concentration using a standard curve (Figure S2B, Supporting Information). This method estimated a concentration of F‐RGD‐DWIVA on the surface of 1215.2 nm, corresponding to a peptide density of 77.4 ± 2.1 pmol cm^−2^. This result is consistent with previous works using catechol‐assisted chemisorption^[^ ^59^ ^]^ or covalent bonding via silanization.^[^ ^70^, ^78^ ^]^ Of note too, such peptide density is well above the minimal concentration required to establish focal contacts, i.e., 10 fmol cm^−2[^ ^79^ ^]^ and integrin‐GF signaling, i.e., 8.9 pmol cm^−2^.^[^ ^59^ ^]^ Finally, Raman spectroscopy was performed to evaluate the distribution of the peptide on Ti surfaces. As shown in Figure S2C (Supporting Information), control Ti did not present any characteristic peaks in the Raman spectrum. However, upon the addition of the RGD‐DWIVA biomolecule several new bands associated to amide groups, in the range of 1300–1700 cm^−1^, were detected. Furthermore, plotting the spectral region between 1400 and 1530 cm^−1^, and mapping an area of the surface of 90 × 80 µm, allowed us to verify a homogenous distribution of the biomimetic peptide on the Ti disks. Taken together, these results demonstrated the successful and homogenous functionalization of Ti with the biomimetic peptides.

### 2.5 Biomimetic Peptides Enhance Human MSCs Adhesion and Promote Larger Vinculin Clusters, Especially When cRGD Motif is Present on the Peptidic Molecule

After verifying the successful functionalization of Ti implants, the biological potential of the substrates modified with the biomimetic peptides was evaluated on human MSCs. To ensure that the biological outcomes were directly related to the activity of the biomimetic peptides, we also engineered two control peptides, in which either the RGD or the DWIVA sequences were mutated (i.e., scrambled versions), to better ascertain the influence of the individual active motifs (peptides coded as DWIVA and RGD conditions, respectively) (see the Experimental Section for details). Prior to evaluating the capacity of the molecules to enhance MSCs adhesion, preliminary studies using the RGD‐DWIVA molecule on model glass coverslips were performed (Figure S3, Supporting Information). In detail, after 7 h in culture with serum‐free medium, cells were fixed and stained for F‐actin (Figure S3A, Supporting Information) to evaluate the number of attached cells and their projected area (Figure S3B,C, Supporting Information). Interestingly, a clear enhancement in MSC adhesion was observed on RGD‐DWIVA functionalized samples in comparison to untreated glass controls. Moreover, a significantly higher number of adherent cells was also observed compared to RGD‐ and DWIVA‐modified surfaces (Figure S3B, Supporting Information), indicating a positive enhancing effect by the copresentation of the two motifs within the biomimetic peptide and in agreement with previous data using C2C12 cells.^[^ ^59^ ^]^ Cell area was also improved in regards to the DWIVA sequence but not to RGD (Figure S3C, Supporting Information). It is also worth mentioning that in a previous study we demonstrated that the copresentation of the individual motifs without controlling their spatial distribution (i.e., random distribution), or the use of immobilized RGD and soluble DWIVA, both failed to support such enhancing effects in cell adhesion. These results clearly illustrate the spatial requirements for integrin‐GF stimulation at the ventral side of the cell and the advantage of using the biomimetic peptide.

**Figure 5:**
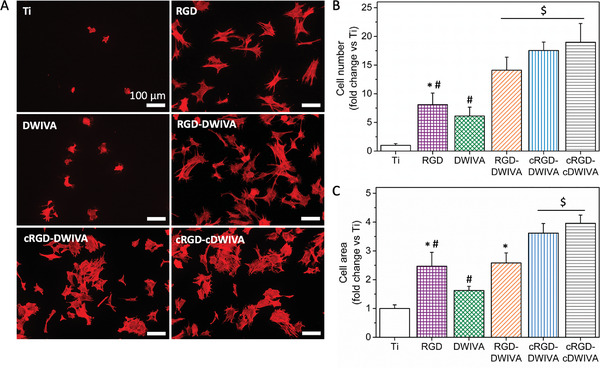
Human MSCs behavior on the functionalized Ti substrates. A) F‐actin immunostaining (scale bar = 100 µm). B) Quantification of cell number and C) cell area after incubating the cells 7 h in serum‐free medium. Each condition was replicated in triplets (*n* = 3) and five pictures per samples were used to quantify cell adhesion. Different symbols denote statistically significant differences between groups (*p* < 0.05).

Subsequently, the RGD‐DWIVA candidate, its cyclic variants (cRGD‐DWIVA and cRGD‐cDWIVA), and the linear controls (RGD and DWIVA) were used to functionalize Ti surfaces and study MSC adhesion (**Figure** **5**). On the conditions presenting the individual peptides, either on the RGD‐ or DWIVA‐coated substrates, cell number and area significantly increased in comparison to the Ti control, in which only a few, roundish cells were attached. Remarkably, the three biomimetic peptides statistically increased the number of cells adhering on the surfaces (Figure 5B), proving the capacity of RGD and DWIVA to engage in positive integrin‐BMPR cross‐signaling when simultaneously presented to cells in a spatially and stoichiometrically controlled manner.^[^ ^59^ ^]^ Although statistical differences were not observed between the three multifunctional peptides, a tendency toward higher cell attachment was shown when cyclization of the RGD peptide was performed (cRGD‐DWIVA and cRGD‐cDWIVA on Figure 5A,B). These results are not surprising, as cyclization improves the activity of the RGD sequence for integrin *α*v*β*3, which is expressed in MSCs^[^ ^62^ ^]^ and involved in the early stages of cell adhesion.^[^ ^61^, ^80^ ^]^ Similar findings were observed in other works when cyclization of RGD was performed.^[^ ^60^, ^81^, ^82^ ^]^ The effects of surface functionalization on cell projected area were generally similar, but with some notable exceptions. As previously observed on model glass surfaces, RGD‐DWIVA increased cell spreading in comparison to uncoated or DWIVA‐functionalized samples, but presented comparable levels as for RGD‐functionalized surfaces (Figure 5A,C). However, in this case, RGD cyclization (i.e., samples cRGD‐DWIVA and cRGD‐cDWIVA) did result in a statistically significant enhancement in cell spreading, compared to all conditions. These results point out, first, to a measurable positive role of DWIVA combined to RGD to improve cell number but not area, and also corroborate the enhancing impact on cell adhesion of cyclic RGD. The cyclization of DWIVA (i.e., cRGD‐cDWIVA) preserved the notable biological responses observed on cRGD‐DWIVA samples. In particular, cDWIVA seemed to slightly improve these effects, but the lack of statistical significance does not allow us to draw further conclusions at this point.

**Figure 6:**
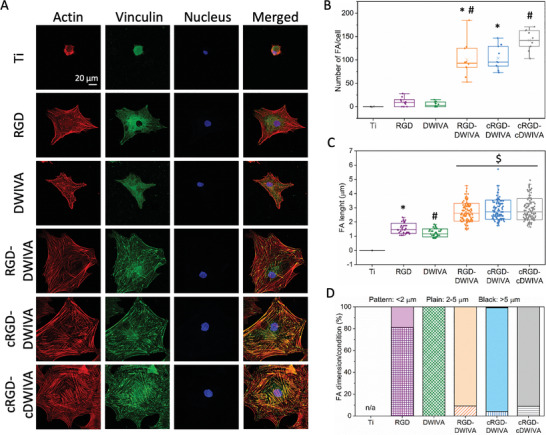
Focal adhesion on Ti disks functionalized with the different peptides after 7 h of culture in serum‐free medium. A) F‐Actin, vinculin and nucleus staining (scale bar = 20 µm). B) Number of focal adhesions per cell and C) focal adhesion length in each condition. D) Vinculin clusters classified as focal complexes (<2 µm), focal adhesions (2–5 µm), and mature adhesions (>5 µm) for each condition. This classification of vinculin clusters is based on previous works.^[^ ^84^, ^85^ ^]^ Each condition was replicated in triplets (*n* = 3) and 100 different focal adhesions were measured to quantify FA lengths. Different symbols denote statistically significant differences between groups (*p* < 0.05).

To further characterize MSC adhesion on the surfaces, focal adhesion (FA) formation was also investigated (**Figure** **6**). As clearly seen by vinculin staining, no focal contacts were observed on Ti control, where cells were totally round. On the RGD‐ or DWIVA‐coated conditions, a few nascent focal complexes were detected (Figure 6A,A). Such focal contacts had a tendency to be slightly larger on the RGD‐coated samples compared to DWIVA (1.55±0.3 µm vs 1.24±0.3 µm, Figure 6C), although no statistical differences were observed. Indeed, on the RGD condition, 80% of focal contacts accounted for focal complexes (<2 µm) and the remaining 20% for focal adhesions (2–5 µm), while on DWIVA, all vinculin clusters were classified as focal complexes, which confirms that RGD supports the assembly of focal adhesions compared to DWIVA (Figure 6D). In contrast, the functionalization of Ti surfaces with either of the three biomimetic peptides was translated into a greater amount of focal adhesions, with a clear shift toward well‐formed and much larger clusters. Although cluster length was similar for the three biomimetic peptides (ranging between 2.68 and 2.90 µm), the condition exposing the two cyclic sequences showed the highest number of focal adhesions (Figure 6B), indicating a positive effect of cyclic DWIVA in stabilizing a higher number of adhesions on the surfaces. Furthermore, in the three biomimetic peptides, most of the focal adhesion (>80%) had a length between 2 and 5 µm, demonstrating the capacity of the biomimetic peptides to induce larger FAs in comparison to the RGD and DWIVA controls. Similar lengths have been observed on flat Ti functionalized with a peptidic platform containing the RGD and PHSRN sequences. In that case, most of the clusters were classified as focal adhesions or super mature adhesions (>5 µm). The observation of more mature adhesions in that work could be explained by the fact that cells were allowed to interact with the functionalized surfaces for a longer period of time than in the present work.^[^ ^83^ ^]^ In another study, with more similar adhesion times, focal adhesions of around 2.2 µm were observed, which is in accordance with our findings.^[^ ^75^ ^]^

MSCs proliferation on the biointerfaces was then assessed after 3, 7, 10, and 17 days of cell culture (Figure S4, Supporting Information). Of note, the differences observed in cell number after 7 h of culture between bare Ti and the rest of the conditions were maintained after 3 days of incubation, which generally indicates the relevance of stimulating integrins and/or BMP receptors in cell proliferation. These differences were less noticeable for longer periods in culture, although uncoated Ti always presented the lowest number of cells. Among the functionalized samples, the DWIVA‐coated Ti displayed the lowest cell proliferation values, in contrast to RGD, which performed the best, especially at day 17. Nonetheless, the differences noticed in the number of attached cells between the RGD‐ and DWIVA‐coated samples in comparison to the three biomimetic peptides (Figure 5) were not observed in cell proliferation (Figure S4, Supporting Information), which indicates that the synergy elicited in cell adhesion with the combination of RGD and DWIVA sequences is not translated in cell proliferation. Interestingly, on day 17 the biomimetic peptides had a tendency to support less cell proliferation than the RGD control, which would be in agreement with a concomitant process of differentiation, as a result of the integrin‐BMPR crosstalk.

### 2.6 Biomimetic Peptides Promote Human MSCs Osteogenic Differentiation

**Figure 7:**
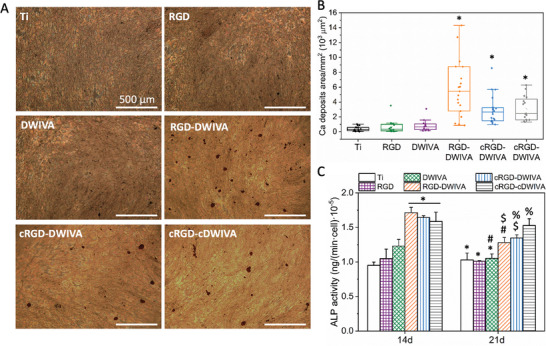
hMSCs biological activity. A) Calcium deposits of hMSCs on functionalized Ti with the different peptides (scale bar = 500 µm). B) Quantification of calcium deposits area. C) ALP activity after 14 and 21 days of culture on the different functionalized surfaces. Each condition was replicated in triplets in each experiment (*n* = 3) and five pictures per sample were used to quantify calcium deposits. Different symbols denote statistically significant differences between conditions (*p* < 0.05).

The capacity of the coatings to promote the osteodifferentiation of MSCs was initially characterized by mineralization and ALP expression studies. In detail, the mineralization of the Ti substrates was evaluated by staining the calcium deposits produced by MSCs after 21 days in culture (**Figure** **7**A). As expected, on Ti surfaces lacking signaling molecules the amount of calcium deposits was marginal and only a few and very small deposits were observed. As shown in Figure 7A and quantified in Figure 7B, mineralization was slightly increased on RGD‐ and especially on DWIVA‐functionalized samples, accounting for the capacity of RGD to interact with integrins *α*v*β*3 and *α*5*β*1,^[^ ^62^ ^]^ which are known to trigger osteogenic differentiation,^[^ ^61^, ^78^ ^]^ and the osteoinductive role of DWIVA,^[^ ^35^ ^]^ respectively. Interestingly, the amount and total area of calcium deposits was clearly increased on the three RGD‐DWIVA biomimetic Ti surfaces (Figure 7A), thus demonstrating a synergistic integrin/GF crosstalk to enhance the osteogenic differentiation of MSCs only when the two sequences are exposed together.

In addition, ALP activity was evaluated after 14 and 21 days of culture (Figure 7C). Well in accordance with the results of mineralization, a significant increase in ALP activity was observed at day 14 when Ti substrates was functionalized with either of the three biomimetic peptides, in comparison with the individual peptide controls and bare Ti. These values decreased at day 21; nevertheless, all biomimetic peptides still presented significant differences with regards to the bare Ti and RGD condition, and the biomimetic peptides containing the cyclic RGD also showed a significant increase in ALP activity with regards to the DWIVA control.

The enhancing effects of combining RGD and DWIVA in ALP expression, and the extent of cell mineralization, were not observed in a previous work by Madl et al. using osteoblasts and MSCs, respectively.^[^ ^39^ ^]^ However, in that study the two sequences were randomly incorporated on alginate hydrogels, and, consequently, the spatial disposition between both peptides was not controlled. Such differences in biological outcomes thus stress the importance of engineering the spatial orientation and stoichiometry as well as the distance between the bioactive sequences in order to effectively elicit synergistic integrin‐GF signaling at the ventral side of the cells, as we recently demonstrated.^[^ ^59^ ^]^

**Figure 8:**
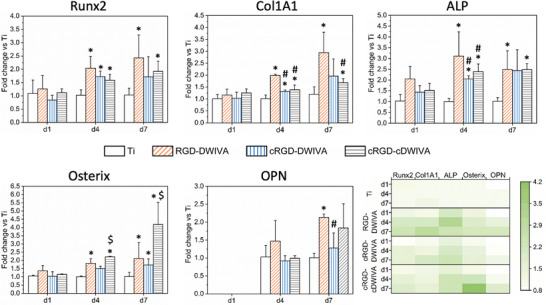
Expression of Runx2, Col1A1, ALP, OPN, and Osterix genes determined by RT‐PCR for human MSCs cultured 1, 4, or 7 days on Ti substrates functionalized with the biomimetic peptides and heatmap of the studied genes representing the relative expression levels. Gene expression was normalized to the housekeeping gene GAPDH. Gene expression is referenced to Ti control at each timepoint. Each condition was replicated in triplets (*n* = 3). * represents statistically significant differences regarding Ti at each time point, # expresses statistically significant differences with respect to RGD‐DWIVA at each time point and $ indicates statistically significant differences with regards to cRGD‐DWIVA at each time point (*p* < 0.05).

To further corroborate the capacity of the molecules to promote osteogenic differentiation at the gene level, gene expression was analyzed at days 1, 4, and 7 by means of RT‐PCR. In particular, Runx2, Col1A1, ALP, Osterix (also known as Sp7) and Osteopontin (OPN) genes, which are involved in osteodifferentiation,^[^ ^86^, ^87^ ^]^ were measured (**Figure** **8**). In the previous biological characterization, the higher biological potential of the multifunctional peptides regarding the scrambled controls (RGD and DWIVA conditions) was clearly demonstrated. Thus, gene expression was only investigated for the biomimetic RGD‐DWIVA peptides and compared with bare Ti. It should also be noted that as OPN is considered a late osteogenic marker,^[^ ^83^, ^88^ ^]^ no measurements were performed at day 1 for this gene.

In general, after 24 h in culture, no significant differences were observed for any of the osteogenic markers, although ALP and Osterix expression on the RGD‐DWIVA condition were higher in comparison to the rest of the biomimetic peptides and Ti control. At day 4, however, an overexpression of the early osteogenic markers (Runx2, Col1A1, ALP, and Osterix genes) was observed on all the functionalized conditions (RGD‐DWIVA, cRGD‐DWIVA, cRGD‐cDWIVA), which was statistically significant in comparison to Ti, except for the Osterix gene on the cRGD‐DWIVA sample. Of note, on the RGD‐DWIVA condition, Runx2, Col1A1, and Osterix showed a twofold increase compared to Ti, whereas the expression of ALP was almost four times higher than Ti. The cRGD‐cDWIVA also promoted a ≥2‐fold increase in the expression of ALP and Osterix genes compared to control Ti. At day 7, all the biomimetic conditions were able to upregulate the expression of the studied genes. In detail, statistical differences were observed for all genes on RGD‐DWIVA, for Osterix on cRGD‐DWIVA, and for all genes except OPN for cRGD‐cDWIVA. These results are summarized in the heatmap represented in Figure 8, in which a higher expression of osteospecific genes is clearly shown for the samples functionalized with the biomimetic peptides, especially at days 4 and 7. The highest increase in gene expression, i.e., 4.2‐fold change versus Ti, is observed for Osterix on the cRGD‐cDWIVA condition at 7 days.

These results are of relevance as differentiation of MSCs toward the osteogenic lineage requires the expression of two main genes: Runx2 and Osterix, both factors involved in the expression of downstream target genes essential for the osteogenic commitment.^[^ ^89^, ^90^ ^]^ In this regard, our results show an overexpression of Runx2 and Osterix for the three biomimetic peptides in comparison to bare Ti at day 4. These results correlate well with our previous mineralization experiments (Figure 7A,B), in which the biomimetic peptides were also able to clearly increase the extent of calcium deposition. Indeed, it has been demonstrated that Runx2 regulates the transcription of osteocalcin, associated with mineralization.^[^ ^91^ ^]^ Of note, the overexpression of Osterix and ALP genes is particularly important in our system, as it has been shown that these two genes are involved in Smad‐independent pathways. In detail, the DWIVA peptide is part of the wrist epitope of the BMP‐2, which interacts with high affinity with BMPR‐I. As a results of such interaction, the activated BMPR‐I recruits BMPR‐II, forming the BMP‐induced signaling complex, and thus, activation of Smad‐independent signaling pathways occurs, triggering overexpression of Osterix and ALP.^[^ ^11^, ^92^, ^93^, ^94^, ^95^ ^]^ In a previous work, we demonstrated that the RGD‐DWIVA peptide activated Smad‐independent signaling through the p38 pathway,^[^ ^59^ ^]^ which is in agreement with the high expression of Osterix and ALP genes observed at days 4 and 7 in the present study.

Furthermore, MSCs osteogenic differentiation in vitro has been associated not only with the increase of ALP activity, but also with the formation of the organic phase of bone ECM, which is mainly composed of collagen type I and noncollagenous proteins like OCN and OPN.^[^ ^96^, ^97^, ^98^ ^]^ Interestingly, overexpression of Col1A1 and ALP genes was observed at days 4 and 7 for the three biomimetic peptides, presenting statistically significant differences in comparison to Ti. However, OPN was only upregulated at day 7, which should be expected taking into account that this protein is considered a late osteogenic marker.^[^ ^88^, ^99^ ^]^

Interestingly, osteogenesis has also been associated with the larger focal adhesions in cells.^[^ ^100^ ^]^ In this regard, greater formation of focal adhesions promotes higher tension of the actin filaments of the cell, thus increasing the tension forces that the cell senses. Consequently, the mechanotransduction phenomenon is initiated, triggering the activation of the Yes‐associated protein (YAP) pathway. Such pathway ultimately activates osteospecific genes, such as Runx2.^[^ ^100^, ^101^, ^102^, ^103^ ^]^ Thus, the overexpression of Runx2 presented by the biomimetic peptides, especially at day 4, may be related to the larger and well‐formed focal adhesions on the three biomimetic substrates (Figure 6), and consequently, having a direct influence on the osteogenic differentiation capacity of the biomimetic peptides. However, further investigations would be required to confirm the correlation between the larger focal adhesions on the biomimetic peptides and its involvement in the mechanotransduction and osteogenic differentiation processes.

Of note, recent works have also achieved regulation of cell adhesion and osteogenesis using non‐BMP‐derived dual peptides. In this regard, Zhu et al. combined a synthetic N‐cadherin peptide with RGD, enhancing the expression of osteogenic gene markers in MSCs and new bone formation and implant osseointegration in vivo.^[^ ^104^ ^]^ Similarly, a Wnt5a mimicking peptide has also been combined with RGD in a stimuli‐responsive nanogel, allowing to synergistically improve cell attachment and osteodifferentiation.^[^ ^105^ ^]^ These examples and our current data illustrate thus the potential of combining synthetic peptide motives to control cell fate at the surface level.

### 2.7 Biomimetic Peptides Enhance New Bone Formation In Vivo

**Figure 9:**
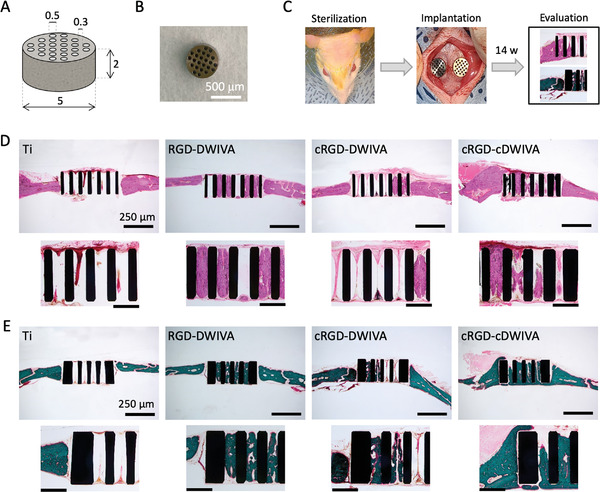
In vivo implantation and staining of the explanted implants. A) Schematic representation of the Ti implant (dimensions are expressed in mm). B) Picture of the Ti implants. C) Schematic representation of the in vivo implantation and histological evaluation. D) Hematoxylin and eosin (H&E) staining of the implants. Scale bar top images = 250 µm. Bottom images are a magnification of the top images at the center of the implant (scale bar = 100 µm). E) Goldner's trichrome (GT) staining of the implants. Scale bar top images = 250 µm. Bottom images are a magnification of the top images at the left side of the implant (scale bar = 100 µm).

**Figure 10:**
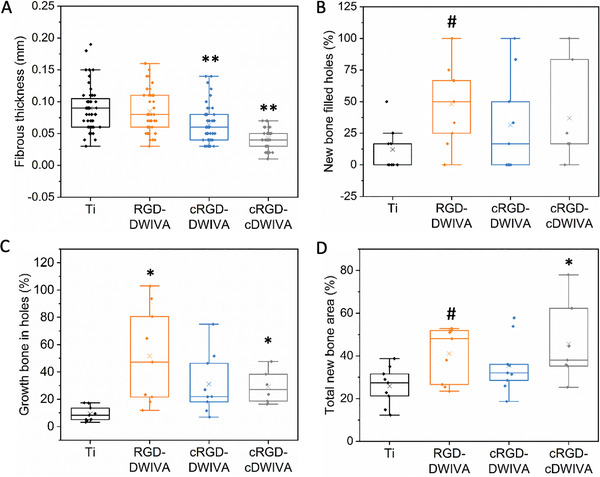
Evaluation of new bone formation in vivo. A) Fibrous thickness around the implants. B) Fraction of the implant holes filled with new bone. C) New bone area fraction on holes normalized to the hole area. D) Total new bone area fraction normalized to the total defect area. Five animals per condition (*n* = 5) were used for the in vivo experiment. ** indicates statistically significant differences versus Ti (*p* < 0.001), * indicates statistically significant differences versus Ti (*p* < 0.05), # indicates statistically significant differences versus Ti (*p* < 0.1).

Having demonstrated the ability of the three biomimetic peptides to induce osteogenic differentiation in vitro, the three molecules were selected to validate their osteoinductive potential in an in vivo scenario. To this end, Ti implants (**Figure** **9**A,B) were functionalized with the three peptides and implanted in a rat calvarial defect. After 14 weeks of implantation, rats were euthanized and the implants and tissues surrounding them were harvested for further analysis (Figure 9C). During the harvesting process, neither visible inflammation nor highly fibrous invasions were observed in any of the samples. Also, the new tissue formed around the implants was stable. Hematoxylin and eosin (H&E) staining (Figure 9D) as well as Goldner's trichrome staining (Figure 9E) were performed to assess new bone formation promoted by the peptide‐coated implant surfaces. Histological examination by H&E staining showed that the implants presented a low number of recruited inflammatory cells (cells with a dark and dense nucleus—purple color staining in Figure S5, Supporting Information), such as eosinophils. In addition, quantification of the fibrous thickness (Figure S5, Supporting Information) around the implants demonstrated the capacity of the biomimetic peptides to reduce fibrous tissue compared to non‐functionalized Ti (**Figure** **10**A), although only the peptides containing cRGD (i.e., cRGD‐DWIVA and cRGD‐cDWIVA) showed a statistically significant decrease (*p* < 0.001). This reduction of fibrous thickness triggered by the biomimetic peptides containing the cyclic RGD in comparison to the linear counterpart may arise from conformational restrictions in cyclic RGD, which increases the affinity toward integrins highly expressed in osteoblasts and MSCs, like *α*v*β*3 integrin. Such specificity toward this particular subtype of integrins may promote a better osseointegration of the implants, thus decreasing the fibrous tissue. Indeed, functionalization of Ti implants with cyclic(RGDfK) showed the capacity to reduce fibrous tissue around the implant as well as to enhance implant fixation and even new bone formation.^[^ ^106^, ^107^, ^108^ ^]^

Following evaluation of fibrous tissue, the fraction of implant holes filled with new bone, area of new bone growth inside the Ti holes, as well as total new bone area fraction, were analyzed (Figure 10B,C,D, respectively). Quantification of the fraction of holes filled with new bone revealed that on bare Ti the implants were not able to support bone growth inside the holes, whereas in the three biomimetic peptides new bone growing inside the holes was observed (Figure 10B), demonstrating the osteogenic capacity of the biomimetic peptides. Although the three biomimetic peptides were able to promote filling of Ti implants with new bone, only the RGD‐DWIVA condition showed significant differences (*p* < 0.1) with regards to nonfunctionalized Ti implants, being the one that presented more filled holes with new bone. Next, new bone area inside the implant holes was normalized to the holes’ space (Figure 10C). Interestingly, the three biomimetic peptides were able to enhance new bone growth inside the holes in comparison to Ti, and the RGD‐DWIVA and cRGD‐cDWIVA conditions presented significant differences. Finally, total new bone area was quantified (Figure 10D). In that case, both the new bone growing inside the holes and the new bone surrounding the implants were considered for analysis. Interestingly, on the functionalized scaffolds thicker new bone grew not only on the area surrounding the implants but also in other zones which were not in direct contact with the implants (Figure S6, Supporting Information). One possible explanation for such phenomena could be the paracrine signal mediated by the biomimetic peptides, which could stimulate new bone growing on the proximity of the implant. Indeed, this effect was observed on Ti cylinders functionalized with *α*v*β*3‐ or *α*5*β*1‐peptidomimetics, which triggered new bone growth underneath of calvarial defects not exposed to the molecules.^[^ ^78^ ^]^ Similar results were obtained when using a fibronectin‐mimetic peptide with increased affinity toward *α*5*β*1 integrins.^[^ ^75^ ^]^

Of note, only cells at the scaffold interface may be stimulated by the biomimetic peptides, so either these cells secrete GFs and stimulate osteogenic differentiation of neighboring cells or the biomimetic peptides could be released from the surface and directly activate other cells. However, the latter hypothesis seems more unlikely to happen, as the catechol‐titanium interaction is very stable, especially in wet conditions.^[^ ^64^, ^109^ ^]^ Nonetheless, more insights would be required to confirm the mechanism of action of the biomimetic peptides in vivo.

Taking all results into consideration, it is clearly demonstrated that the biomimetic peptides improved new bone formation in vivo in comparison to nonfunctionalized Ti, thus proving the osteogenic capacity of combining integrin and GF signaling and confirming the results obtained in the in vitro experiments with MSCs. In particular, the biomimetic peptides RGD‐DWIVA and cRGD‐cDWIVA displayed the highest values of new bone formation, which is in agreement with the PCR results. It would seem, therefore, that although introducing cyclic RGD in the biomimetic peptides has an enhancing effect in cell adhesion and focal adhesion formation compared to its linear analogue, such positive effect is not translated in terms of osteogenic differentiation or new bone formation in vivo. This would imply that engaging in integrin‐GF signaling is necessary to increase the osteodifferentiation of MSCs but that an increase in the affinity for *α*v*β*3 cannot be correlated with higher bone formation in vivo.

## 3 Conclusion

In conclusion, in this work we identified and designed peptides derived from the wrist and knuckle epitopes of BMP‐2 and screened their osteogenic potential taking advantage of the C2C12 transdifferentiation capacity toward the osteogenic lineage. No sequences with better osteogenic capacity than the DWIVA peptide were found. It would be thus interesting to further explore high‐throughput optimization techniques, such as bioinformatics and in silico modeling, to find other peptidic candidates with osteogenic potential. Nonetheless, the combination of the DWIVA motif (and its cyclic counterpart) with integrin‐binding sequences in biomimetic multifunctional peptides demonstrated the capacity of such peptides to simultaneously stimulate integrin and BMP receptors, as shown by the clear enhancement of MSCs adhesion, osteogenic differentiation, and in vivo bone formation in comparison to the controls. Importantly, this strategy shows the capacity of synthetic peptides to install osteogenic and osteoconductive properties on clinically relevant materials, such as Ti. Advancing in novel strategies that could potentially replace the use of BMP‐2 holds promise to overcome the negative side effects associated to the use of GFs. Moreover, the enhancing effects of cyclic RGD in cell adhesion, compared to linear RGD, when combined with the DWIVA motif, were not translated in terms of osteogenic differentiation or new bone formation in vivo. In this regard, further investigations to better understand the integrin‐BMP receptor crosstalk and signaling pathways triggered by the biomimetic peptides would be required. In addition, correlating such BMP‐signaling events with mechanotransduction phenomena would be also necessary, as it has been demonstrated the association between BMP‐2 signaling and mechanotransduction through the YAP/TAZ pathway.^[^ ^110^ ^]^ Furthermore, the effect of osteogenic and cell adhesive peptides in bone immunoregulation and metabolism has been recently studied.^[^ ^111^, ^112^, ^113^ ^]^ Thus, the investigation of such processes in our systems would be also interesting. Finally, the translation of this strategy from 2D to 3D biomaterials, such as hydrogels, to study cell behavior on 3D microenvironments mimicking bone ECM, and comparing their potential with the administration of BMP‐2 is warranted.

## 4 Experimental Section

### Peptide Synthesis and Characterization—Chemical Reagents and Instrumentation—Reagents

Fmoc‐Rink amide MBHA resin, Fmoc‐2‐CTC‐OH‐PS resin and Fmoc‐*L*‐amino acids were purchased from Iris Biotech GmbH (Germany) and Novabiochem—Merck KGaA (Germany). Fmoc‐*L*‐DOPA(acetonide)‐OH was obtained from Bachem (Switzerland). Coupling reagents and additives were obtained from Sigma‐Aldrich (USA), and Iris Biotech GmbH and Novabiochem—Merck KGaA. All other chemicals and solvents were acquired from Sigma‐Aldrich and Carlo Erba (Spain).

### Chemical Reagents and Instrumentation—Reversed‐Phase Analytical High‐Performance Liquid Chromatography (RP‐HPLC)

HPLC analysis was performed using a Shimadzu Prominence XR equipped with a LC‐20AD pump, a SIL‐20AC cooling autosampler, a CTO‐10AS column oven, and a SPD‐M20A photodiode array detector (Shimadzu, Japan). A reversed‐phase XBridge (Waters, USA) C18 column (4.6 × 100 mm^2^, 3.5 µm) column was used. The system was run at a flow rate of 1.0 mL min^−1^ over 8 min at room temperature (RT) using water (0.045% trifluoroacetic acid (TFA), v/v) and acetonitrile (ACN) (0.036% ACN, v/v) as mobile phases with UV detection at 220 nm.

### Chemical Reagents and Instrumentation—Semipreparative RP‐HPLC Purification

purification was performed using a Shimadzu instrument equipped with a LC‐8A pump and a photodiode array detector (Shimadzu, Japan). A reversed‐phase C18 column (10 × 250 mm^2^, 10 µm) (Phenomenex, USA) was used. The system was run at a flow rate of 5.0 mL min^−1^ over 30 min at room temperature using water (0.1% TFA, v/v) and ACN (0.1% ACN, v/v) as mobile phases with UV detection at 220 nm.

### Chemical Reagents and Instrumentation—Matrix‐Assisted Laser Desorption Ionization–Time of Flight (MALDI‐TOF)

It was performed on an Applied Biosystems/MDS SCIEX 4800 Plus with a N_2_ laser of 337 nm using *α*‐cyano‐4‐hydroxycinnamic acid (ACH) matrix (10 mg mL^−1^ of ACH in ACN/H_2_O (1:1, v/v) containing 0% TFA).

### Linear Peptides—General Protocols of Peptide Synthesis

Linear peptides were manually synthesized by SPPS following the Fmoc/tBu strategy and using Fmoc Rink‐amide MBHA resin (0.45 mmol g^−1^) as solid support. For each synthesis polypropylene syringes equipped with polyethylene filters were used. Solvents and soluble reagents were removed using a vacuum filtration system. Washings between couplings and deprotection steps were carried out with *N*,*N*‐dimethylformamide (DMF) and dichloromethane (DCM) (3 times each) using 10 mL of solvent g^−1^ of resin each time. Peptide synthesis was performed at RT. Couplings were monitored using the Ninhydrin test and RP‐HPLC analysis.

### Linear Peptides—Fmoc Group Removal

The Fmoc group was removed by treatment with piperidine/DMF (20:80, v/v) solution (1 × 1 min, 2 × 5 min).

### Linear Peptides—Amino Acid Coupling

Amide bond formation was carried out with Fmoc‐*L*‐amino acids (5 eq.), ethyl 2‐cyano‐2‐(hydroxyimino)acetate (OxymaPure) (5 eq.), and *N*,*N*‐diisopropylcarbodiimide (DIPCDI) (5 eq.) in DMF for 30 min.

### Linear Peptides—N‐*α* Acylation

Acetylation of the *N*‐terminus was carried out with Ac_2_O/DIEA/DMF (10:20:70, v/v/v) (1 × 5 min, 2 × 10 min).

### Linear Peptides—Cleavage and Sidechain Deprotection

The peptidyl‐resin was washed with DMF (5 × 0.5 min) and DCM (5 × 0.5 min) and treated with TFA/H_2_O/triisopropylsilane (TIS) (95:2.5:2.5, v/v/v) for 1 h. Afterward, the excess of TFA was evaporated with a stream of N_2_, and the peptide was precipitated with ice‐cold diethyl ether (Et_2_O) and centrifuged (twice). The crude peptide was dissolved in H_2_O/ACN (1:1, v/v) and lyophilized.

### Linear Peptides—Peptide Characterization

All peptides were purified by semipreparative RP‐HPLC. The purified peptides were characterized by analytical RP‐HPLC and MALDI‐TOF as follows:

**DWIVA** (Ac‐Asp‐Trp‐Ile‐Val‐Ala‐NH_2_): RP‐HPLC (20‐100% ACN over 8 min, *t*_R_ = 5.69 min, purity 99%), MALDI‐TOF (m/z): [M+Na]^+^ Calcd. for C_31_H_45_N_7_O_8_, 666.33; found, 666.35.

**Knuckle** (Ac‐Lys‐Ile‐Pro‐Lys‐Ala‐Ser‐Ser‐Val‐Pro‐Thr‐Glu‐Leu‐Ser‐Ala‐Ile‐Ser‐Thr‐Leu‐Tyr‐Leu‐NH_2_): RP‐HPLC (20‐90% ACN at 60 °C over 8 min, *t*_R_ = 6.41 min, purity 99%), MALDI‐TOF (m/z): [M+H]^+^ Calcd. for C_99_H_167_N_23_O_30_, 2158.22; found, 2159.26.

**WNDWIVA** (Ac‐Trp‐Asn‐Asp‐Trp‐Ile‐Val‐Ala‐NH_2_): RP‐HPLC (20–100% ACN over 8 min, *t*_R_ = 6.61 min, purity 99%), MALDI‐TOF (m/z): [M+Na]^+^ Calcd. for C_46_H_61_N_11_O_11_, 966.46; found, 966.49.

**3Ahx** (Ac‐Trp‐Asn‐Asp‐Trp‐Ile‐Val‐Ala‐(Ahx)_3_‐Leu‐Ala‐Asp‐NH_2_): RP‐HPLC (30‐100% ACN at 60 °C over 8 min, t_R_ = 5.52 min, purity 80%), MALDI‐TOF (m/z): [M+Na]^+^ Calcd. for C_77_H_115_N_17_O_19_, 1604.86; found, 1604.89.

**2Ahx** (Ac‐Trp‐Asn‐Asp‐Trp‐Ile‐Val‐Ala‐(Ahx)_2_‐Leu‐Ala‐Asp‐NH_2_): RP‐HPLC (20–100% ACN at 60 °C over 8 min, *t*_R_ = 6.76 min, purity 76%), MALDI‐TOF (m/z): [M+Na]^+^ Calcd. for C_71_H_104_N_16_O_18_, 1491.77; found, 1491.80.

**1Ahx** (Ac‐Trp‐Asn‐Asp‐Trp‐Ile‐Val‐Ala‐Ahx‐Leu‐Ala‐Asp‐NH_2_): RP‐HPLC (20–100% ACN over 8 min, *t*_R_ = 6.79 min, purity 86%. MALDI‐TOF (m/z): [M+Na]^+^ Calcd. for C_65_H_93_N_15_O_17_, 1378.69; found, 1378.70.

**cDWIVA** (cyclic(Asp‐Trp‐Ile‐Val‐Ala))

2‐Chlorotrityl chloride resin (576 mg, 0.7 mmol g^−1^) was placed in a propylene syringe. The first amino acid, Fmoc‐*L*‐Ala‐OH (125.5 mg, 0.4 mmol, 0.7 eq.), was introduced using DIEA (7 eq.) for 1 h in DCM. The excess of reactive positions was capped with 1.3 mL of MeOH in DMF for 10 min. Fmoc was removed using piperidine in DMF (20:80, v/v) (1 × 1, 1 × 5, 1 × 10 min) and the second residue, Fmoc‐*L*‐Val‐OH (410.53 mg, 1.2 mmol, 3 eq.), was coupled using OxymaPure (3 eq.) and DIPCDI (3 eq.) for 1 h in DMF. Then, the peptide chain (*L*‐Asp(tBu)‐Trp(Boc)‐*L*‐Ile) was elongated using standard Fmoc/tBu strategy. After removal of the Fmoc group, the peptide cleavage was carried out by treating the resin 6 times with TFA/DCM (2:98, v/v) for 30 s. The filtrate was collected over H_2_O, concentrated under N_2_ and precipitated onto ice‐cold Et_2_O. The crude was dissolved in H_2_O/ACN (1:1, v/v) and lyophilized to give 273 mg of crude linear peptide (99% purity).

164 mg of linear peptide was dissolved in 0.4 mm tetrahydrofuran (THF)/DMF (95.5: 0.5, v/v) in a round‐bottom flask. PyBOP (2 eq.), HOAt (2 eq.), and DIEA (4 eq.) were added and the pH was adjusted with DIEA until pH 7. The reaction mixture was stirred at RT for 1 h. After that time, THF was evaporated under vacuo and coevaporated with DCM until dryness. The side‐chain protecting groups removal was performed by treating the crude with TFA/TIS/H_2_O (95:2.5:2.5, v/v/v) (20 mL for 1.5 h). The crude was concentrated and precipitated onto ice‐cold Et_2_O. The crude was dissolved in H_2_O/ACN (1:1, v/v) and lyophilized to give 67.9 mg of crude peptide (83% purity) and it was used without further purification.

Characterization: RP‐HPLC (linear gradient from 20% to 100% (0.036% TFA in ACN/0.045% TFA in H_2_O) in 8 min; *t*_R_ = 7.99 min, 83% purity).

### Synthesis of Protected Cyclic Building Blocks—Cyclic(Arg(Pbf)‐Gly‐Asp(tBu)‐D‐Phe‐Glu(OH))

The cyclic peptide was synthesized using a 2‐chlorotrityl chloride resin (1.5 g, 0.7 mmol g^−1^), as recently reported.^[^ ^60^ ^]^ In brief, the first amino acid, Fmoc‐*L*‐Gly‐OH, was loaded, followed by a capping of the excess of reactive positions of the resin. Fmoc was removed and the second residue, Fmoc‐*L*‐Arg(Pbf)‐OH was introduced. Then, the peptide chain (Asp(tBu)‐*D*‐Phe‐Glu(OAll)) was elongated using standard Fmoc/tBu chemistry. After removal of the Fmoc group, the peptide cleavage was performed and the filtrate was collected, concentrated, and precipitated. The crude peptide was dissolved in H_2_O/ACN (1:1, v/v) and lyophilized to give 887 mg of crude linear peptide (97% purity).

The linear peptide was then cyclized used PyBOP chemistry. After 1 h reaction, the organic layer was extracted, dried over MgSO_4_, filtered, and concentrated under vacuo to give an oily crude. Finally, the Allyl group was removed by treatment with Pd(PPh_3_)_4_‐PhSiH_3_ (0.1 and 8 eq., respectively) in DCM under N_2_ atmosphere for 1 h, followed by evaporation of DCM under vacuo and purification of the crude with a PoraPak Rxn RP 20 cc cartridge using a H_2_O/ACN gradient (0–100%) to remove Pd traces. The product was lyophilized to give 410 mg of peptide.

Characterization: RP‐HPLC (linear gradient from 40% to 100% (0.036% TFA in ACN/0.045% TFA in H_2_O) in 8 min; *t*_R_ = 5.85 min, 89% purity). MALDI‐TOF (m/z): [M+H]^+^ Calcd. for C_43_H_60_N_8_O_12_S 913.41; found, 913.49.

### Synthesis of Protected Cyclic Building Blocks—Cyclic(Asp(tBu)‐Trp(Boc)‐Ile‐Val‐Ala‐Glu(OH))

2‐Chlorotrityl chloride resin (1.6 g, 0.7 mmol g^−1^) was placed in a propylene syringe. The first amino acid, Fmoc‐*L*‐Ala‐OH (350.1 mg, 1.1 mmol, 0.7 eq.), was loaded using DIEA (7 eq.) for 1 h in DCM. The excess of reactive positions was capped with 1.3 mL of MeOH in DMF for 30 min. Fmoc was removed using piperidine in DMF (20:80, v/v) (1 × 1, 1 × 5, 1 × 10 min) and the second residue, Fmoc‐*L*‐Val‐OH (1145.1 mg, 3.4 mmol, 3 eq.), was incorporated using OxymaPure (3 eq.) and DIPDCI (3 eq.) for 1 h in DMF. Then, the peptide chain (*L*‐Glu(OAll)‐*L*‐Asp(tBu)‐*L*‐Trp(Boc)‐*L*‐Ile) was elongated using standard Fmoc/tBu chemistry. After removal of the Fmoc group, the peptide cleavage was performed by treating the resin 6 times with TFA/DCM (2:98, v/v) for 30 s. The filtrate was collected over H_2_O, concentrated under N_2_ and precipitated onto ice‐cold Et_2_O. The crude was dissolved in H_2_O/ACN (1:1, v/v) and lyophilized to give 479 mg of crude linear peptide (90% purity).

250.3 mg of linear peptide was dissolved in 0.4 mm THF/DMF (95.5: 0.5, v/v) in a round‐bottom flask. PyBOP (2 eq.), HOAt (2 eq.), and DIEA (4 eq.) were added and the pH was adjusted with DIEA until pH 7. The reaction mixture was stirred at RT for 30 min. Then, the Allyl group was removed by treatment with Pd(PPh_3_)_4_‐PhSiH_3_ (0.1:8) in DCM under the N_2_ atmosphere for 1 h. After that time, DCM was evaporated under vacuo and the crude was purified with a PoraPak Rxn RP 20 cc cartridge using a H_2_O/ACN gradient (0–100%) to remove Pd traces. Finally, the product was lyophilized to give 260 mg of peptide.

Characterization: RP‐HPLC (linear gradient from 20% to 100% (0.036% TFA in ACN/0.045% TFA in H_2_O) in 8 min at 60 °C; *t*_R_ = 6.91 min, 55% purity).

### Biomimetic Peptides

**cRGD‐DWIVA** *[(cyclic(Arg‐Gly‐Asp‐D‐Phe‐Glu)‐Ahx‐Ahx)(Ac‐Asp‐Trp‐Ile‐Val‐Ala‐Ahx‐Ahx)]‐Lys‐βAla‐DOPA‐DOPA‐NH_2_*

Fmoc–Rink Amide MBHA resin (136.5 mg, 0.4 mmol g^−1^) was placed in a propylene syringe. Fmoc was removed using piperidine in DMF (20:80, v/v) (1 × 1, 1 × 5, 1 × 10 min), and then Fmoc‐*L*‐DOPA(acetonide)‐OH (31.4 mg, 0.07 mmol, 0.5 eq.) was incorporated using OxymaPure (0.5 eq.) and DIPCDI (0.5 eq.) for 1 h 30 min in DMF. The excess of reactive positions was capped with Ac_2_O (5 eq.) and DIEA (5 eq.) in DMF for 30 min. After removal of the Fmoc group, the second Fmoc‐*L*‐DOPA(acetonide)‐OH (93.7 mg, 0.2 mmol, 1.5 eq.), was incorporated using the same methodology. Then, the peptide chain ((Fmoc‐Ahx‐OH)_2_‐Lys(Alloc)‐*β*Ala) was elongated using standard Fmoc/tBu chemistry.

Fmoc was removed using piperidine in DMF (20:80, v/v) (1 × 1, 1 × 5, 1 × 10 min), and then *cyclic(Arg(Pbf)‐Gly‐Asp(tBu)‐D‐Phe‐Glu(OH))* (100 mg, 0.10 mmol, 2 eq.) was incorporated using PyBOP (4 eq.), HOAt (4 eq.), and DIEA (8 eq.) at pH 8 for 1 h in DMF. Then, the resin was washed, and a recoupling was done using the same amount of eq. The few unreactive positions were capped with Ac_2_O (5 eq.) and DIEA (5 eq.) in DMF for 30 min. Then, the Alloc group was first removed using catalytic amounts of palladium. The resin was then washed with a solution of sodium diethyldithiocarbamate (0.02 m in DMF; 3 × 15 min) and the 2Ahx were coupled in the second peptide chain. Then, the second peptide chain (DWIVA) was elongated using standard Fmoc/tBu chemistry and the N‐terminus acetylated by treatment with Ac_2_O/DIEA/DMF (1:2:7, v/v/v) (1 × 5, 2 × 10 min).

The cleavage and deprotection was performed by treating the resin with TFA/TIS/H_2_O (95:2.5:2.5, v/v/v) (5 mL for 1.5 h). The filtrate was collected, concentrated, and precipitated onto ice‐cold Et_2_O. The crude was dissolved in H_2_O/ACN (1:1, v/v) and lyophilized to give 60 mg of crude peptide (42% purity). Peptide **cRGD‐DWIVA** was isolated by semipreparative RP‐HPLC, and 6.7 mg was obtained.

Characterization: RP‐HPLC (linear gradient from 25% to 80% (0.036% TFA in ACN/0.045% TFA in H_2_O) in 8 min; *t*_R_ = 5.90 min, 80% purity). MALDI‐TOF (m/z): [M+H]^+^ Calcd. for C_108_H_159_N_24_O_28_ 2239.17; found, 2240.10.

**cRGD‐cDWIVA** *[(cyclic(Arg‐Gly‐Asp‐D‐Phe‐Glu)‐Ahx‐Ahx)(cyclic(Asp‐Trp‐Ile‐Val‐Ala‐Glu)‐Ahx‐Ahx)]‐Lys‐βAla‐DOPA‐DOPA‐NH_2_*

Fmoc–Rink Amide MBHA resin (147 mg, 0.4 mmol g^−1^) was placed in a propylene syringe. Fmoc was removed using piperidine in DMF (20:80, v/v) (1 × 1, 1 × 5, 1 × 10 min), and then then Fmoc‐*L*‐DOPA(acetonide)‐OH (34 mg, 0.07 mmol, 0.5 eq.) was incorporated using OxymaPure (0.5 eq.) and DIPCDI (0.5 eq.) for 1 h 30 min in DMF. The excess of reactive positions was capped with Ac_2_O (5 eq.) and DIEA (5 eq.) in DMF for 30 min. After removal of the Fmoc group, the second Fmoc‐*L*‐DOPA(acetonide)‐OH (40.5 mg, 0.09 mmol, 1.5 eq.), was incorporated using the same methodology. Then, the peptide chain ((Fmoc‐Ahx‐OH)_2_‐Lys(Alloc)‐*β*Ala) was elongated using standard Fmoc/tBu chemistry.

Fmoc was removed using piperidine in DMF (20:80, v/v) (1 × 1, 1 × 5, 1 × 10 min), and then *cyclic(Arg(Pbf)‐Gly‐Asp(tBu)‐D‐Phe‐Glu(OH))* (134.2 mg, 0.15 mmol, 2.5 eq.) was incorporated using PyBOP (4 eq.), HOAt (4 eq.), and DIEA (8 eq.) at pH 8 for 45 min in DMF. Then, the resin was washed, and a recoupling was done using the same amount of eq. Then, the side‐chain protecting group of Lys, Alloc group, was deprotected using catalytic amounts of palladium. The resin was then washed with a solution of sodium diethyldithiocarbamate (0.02 m in DMF; 3 × 15 min) and the 2Ahx were coupled in the second peptide chain. Then, compound *cyclic(Asp(tBu)‐Trp(Boc)‐Ile‐Val‐Ala‐Glu(OH))* (261 mg, 0.3 mmol, 3 eq.) was incorporated using PyBOP (4 eq.), HOAt (4 eq.), and DIEA (8 eq.) at pH 8 for 2 h in DMF. Then, the resin was washed, and a recoupling was done using the same amount of eq. The cleavage and deprotection was performed by treating the resin with TFA/TIS/H_2_O (95:2.5:2.5, v/v/v) (5 mL for 1.5 h). The filtrate was collected, concentrated, and precipitated onto ice‐cold Et_2_O. The crude was dissolved in H_2_O/ACN (1:1, v/v) and lyophilized to give 56.3 mg of crude peptide (36% purity). Peptide **cRGD‐cDWIVA** was isolated by semipreparative RP‐HPLC, and 10.3 mg was obtained.

### Characterization

RP‐HPLC (linear gradient from 20% to 100% (0.036% TFA in ACN/0.045% TFA in H_2_O) in 8 min; *t*_R_ = 6.58 min, 97% purity). MALDI‐TOF (m/z): [M+H]^+^ Calcd. for C_111_H_161_N_25_O_29_ 2308.19; found, 2309.14.

**RGD‐DWIVA** *[(Ac‐Arg‐Gly‐Asp‐Ser‐Ahx‐Ahx)(Ac‐Asp‐Trp‐Ile‐Val‐βAla‐Ahx‐Ahx)]‐Lys‐Ala‐DOPA‐DOPA‐NH_2_*; **RGD** *[(Ac‐Arg‐Gly‐Asp‐Ser‐Ahx‐Ahx)(Ac‐Trp‐Asp‐Ala‐Ile‐Val‐Ahx‐Ahx)]‐Lys‐βAla‐DOPA‐DOPA‐NH_2_*; **DWIVA** *[(Ac‐Arg‐Asp‐Gly‐Ser‐Ahx‐Ahx)(Ac‐Asp‐Trp‐Ile‐Val‐Ala‐Ahx‐Ahx)]‐Lys‐βAla‐DOPA‐DOPA‐NH_2_*

The detailed synthesis and characterization of the biomimetic peptide **RGD‐DWIVA** and the scrambled controls (**RGD** and **DWIVA**) have been previously published.^[^ ^59^ ^]^

In summary, the peptide syntheses were carried out manually by SPPS using Fmoc/tBu strategy and Fmoc‐Rink‐amide MBHA resin (200 mg, 0.74 mmol g^−1^). The Fmoc protecting group was removed by treatment with piperidine/DMF (20:80, v/v) solution (1 × 1, 2 × 5 min) and the Alloc group using catalytic amounts of palladium. Amino acid couplings were carried out with Fmoc‐*L*‐AA‐OH (4 eq.), OxymaPure (4 eq.), and DIPCDI (4 eq.) in DMF for 90 min. For the acetylation of the N‐terminus, a solution of Ac_2_O/DIEA/DMF (10:20:70, v/v/v) (1 × 1 min, 2 × 5 min) was used. The peptide cleavage was carried out treating the peptidyl‐resin with harsh acidic conditions; TFA/H_2_O/TIS (95:2.5:2.5, v/v/v) for 3 h, followed by standard work up and HPLC purification.

### Surface Functionalization

Titanium (Ti) disks (10 mm diameter) of commercially pure (CP) grade 2 Ti were obtained by turning from a cylindrical bar (Harald Pihl, Sweden). Ti disks were smoothed with P600, P1200, and P2500 SiC grinding papers (Neuertek S.A., Spain) and polished with a suspension of silica particles (0.06 µm particle size) on cotton clothes until achieving mirror‐like surfaces. Afterward, samples were cleaned in a sonication bath of cyclohexane, isopropanol, Milli‐Q water, ethanol, and acetone three times for 5 min each, followed by a drying process with N_2_ gas. Subsequently, a 100 µL drop containing the peptidic molecules at a 100 µm concentration in distilled water was deposited on top of the disks and incubated overnight at RT. After this time, the remaining peptide solution was removed and samples were washed three times with distilled water and dried with nitrogen gas. Prior to cell assays, all samples were blocked with 1% bovine serum albumin (BSA) in phosphate buffered saline (PBS) w/v for 30 min to avoid nonspecific interactions between the cells and the surfaces. Next, samples were washed twice with PBS and sterilized with 70% ethanol v/v for 5 min, washed again twice with PBS and finally placed in sterile 48‐well plates. The bottom of each well was previously blocked with 1% BSA solution. Note that the different conditions in the study were coded according to the peptidic molecule used to functionalized Ti substrates. For instance, RGD denotes Ti disks functionalized with the RGD peptidic molecule. The same nomenclature was used for the rest of conditions. Bare Ti without functionalization was used as a negative control (Ti).

Round glass cover slips (10 mm diameter) were washed with distilled water, ethanol, and acetone (three times for 5 min each), followed by a sterilization process with 70% ethanol v/v for 5 min and washed then three times with PBS. For the selection of the sequences with osteogenic potential, peptides were physically adsorbed on glass surfaces by depositing 200 µL of 100 µm peptide solution, incubated overnight at RT and left to dry under sterile conditions. For preliminary cell adhesion studies, the same protocol as for Ti disks was used to functionalize glass substrates. Bare glass without functionalization was used as a control (Glass).

### Physicochemical Surface Characterization—Surface Chemical Composition

Detection of C, O, N, and Ti elements was conducted on an X‐ray photoelectron spectroscopy system (SPECS XPS System, Berlin, Germany), which was equipped with a XR50 Al anode operating at 150 W (10 kV), a hemispherical analyzer (Phoibos 150), and a MCD‐9 electron detector. The electron takeoff angle was set to 90° relative to the sample surface and the pass energy was fixed at 20 eV, with 0.1 eV steps (high resolution) or 1 eV steps (survey spectra). The working pressure was below 5 × 10^−8^ mbar. Casa XPS software (Version 2.3.19PR1.0, Casa Software Ltd, UK) was used to analyze the data. Prior to analysis, C 1s spectra were calibrated at 284.4 eV and the rest of the peaks were referenced to such energy.

### Physicochemical Surface Characterization—Surface Wettability

The hydrophilicity of the samples was measured by static contact angle. Measurements were performed using a Contact Angle System OCA15 plus (DataPhysics, Germany) and Milli‐Q water as wetting liquid (drop volume of 1 µL). Three measurements were performed per sample, using three replicates per condition. Contact angle values were obtained using a Laplace–Young fitting with SCA 20 software (DataPhysics, Germany).

### Physicochemical Surface Characterization—Surface Peptide Detection

Raman spectroscopy was used to analyze characteristic functional groups related to the presence of the biomolecule. Raman spectra were obtained using a Renishaw inVia Qontor confocal Raman microscope (Renishaw, UK) with a 532 nm excitation laser source with 2400 L mm^−1^ and using a 20x objective with laser power of 50 mm (10%) and an exposure time of 1 s. For the mapping measurements, a random area of 90 × 80 µm^2^ was selected and the measurement step was set at 10 µm. Data was processed with WiRE 4.4 software (Renishaw, UK).

### Physicochemical Surface Characterization—Peptide Quantification

The peptide surface density on Ti was assessed according to a previously published protocol.^[^ ^59^ ^]^ Briefly, functionalized Ti with F‐RGD‐DWIVA was treated with 100 µL of 1 m NaOH at 70 °C for 12 min. Afterward, 50 µL of the hydrolysate were added to a 96‐well plate with black bottom. Simultaneously, a calibration curve of well‐known F‐RGD‐DWIVA concentrations (from 0 to 2000 nm) was performed. 50 µL of each concentration of the standard curve were also added to the 96‐well plate with black bottom. Finally, fluorescence intensity was quantified (*λ*_excitation_ = 485 nm, *λ*_emission_ = 528 nm) with a microplate reader (Infinite M200 PRO, Tecan Group Ltd., Switzerland). Conversion of the fluorescence intensity readout from functionalized samples into concentration of F‐RGD‐DWIVA was performed by plotting such value in the standard curve.

### In Vitro Biological Characterization—Cell Culture

C2C12 mouse myoblasts (ATCC, USA) were cultured in Dulbecco's Modified Eagle Medium (DMEM) (Gibco, USA) with D‐glucose, sodium pyruvate, and supplemented with 10% v/v FBS (BMP‐2 free), 2 mm *L*‐glutamine and penicillin/streptomycin (50 U mL^−1^ and 50 µg mL^−1^, respectively). Upon reaching 60–70% confluence, cells were detached with Accutase and plated in new flasks. C2C12 were used between passage 2 and 8. Human bone marrow mesenchymal stem cells (MSCs) (ATCC, USA) were cultured in Advanced Dulbecco's Modified Eagle Medium (Adv. DMEM) with D‐glucose, nonessential amino acids, sodium pyruvate, and supplemented with 10% FBS v/v, 20 mm HEPES, 2 mm *L*‐glutamine, and penicillin/streptomycin (50 U mL^−1^ and 50 µg mL^−1^, respectively). Cells were detached with trypsin‐EDTA and plated in new flasks when they reached 70–80% confluence. MSCs were used between passages 4 and 6. All cells were maintained at 37 °C in a humidified atmosphere with 5% of CO_2_, replacing culture media every 2 days. Cell density and passage are indicated in each particular experiment.

### In Vitro Biological Characterization—C2C12 Myotube Area Quantification

C2C12 cells at passage 8 were maintained for 4 h in DMEM‐1% FBS. Afterwards, 20 000 cells per well were seeded onto functionalized or nonfunctionalized glass substrates in DMEM‐2% FBS. After 6 h, medium was changed and fresh low‐serum medium was added (control conditions). 100 µm of soluble peptide were also added to the nonfunctionalized glasses (peptide‐soluble conditions). Soluble (20 nm) and adsorbed (150 nm) BMP‐2 were used as controls. After 3 days, medium was refreshed without further stimulation. On day 6, cells were fixed with 4% paraformaldehyde (PFA) in PBS v/v for 30 min, permeabilized with 0.05% v/v Triton X‐100 in PBS for 20 min and blocked with 1% BSA w/v in PBS for 30 min. Afterward, myotubes were stained with monoclonal antimyosin heavy chain (14‐6503‐82, Invitrogen, USA) (1:250) in BSA 1% for 2 h, followed by Alexa 488 antimouse IgG antibody (A28175, Invitrogen, USA) (1:2000) in 0.05% Triton for 1 h. Nuclei were stained with 4’,6‐diamidino‐2‐phenyldole (DAPI) (D9542, Sigma‐Aldrich, USA) (1:1000) in PBS‐Glycine for 5 min. Washing between treatments was done with PBS‐Glycine (three times for 5 min each). Samples were then mounted on a microscope slide and imaged using an AF7000 fluorescence inverted microscope (Leica, Germany). Fiji/Image‐J (Image‐J, USA)^[^ ^114^ ^]^ was used to quantify the number of nuclei as well as the myosin projected area of the cells.

### In Vitro Biological Characterization—C2C12 ALP Activity

C2C12 cells at passage 2 were cultured for 4 h in DMEM‐1% FBS. Then, 7500 cells/per well were seeded on functionalized glass as described before. After incubating the cells 8 days as indicated in the previous experiment, they were rinsed twice with PBS and lysed with mammalian protein extraction reagent (M‐PER) (Thermo Fisher Scientific, USA). Alkaline phosphatase (ALP) activity was then quantified using the SensoLyte pNPP Alkaline Phosphatase Activity Kit (AnaSpech Inc., USA). In brief, cells were incubated for 1 h at 37 °C with the reagents described in the kit protocol. After stopping the reaction, ALP levels were obtained by measuring the absorbance at 405 nm using a Synergy HTX multimode reader (Bio‐Tek, USA). For each condition, ALP activity was normalized to cell number, which was measured by quantifying the released lactate dehydrogenase (LDH) using the Cytotoxicity Detection kitPLUS (LDH) (Roche, USA). After 7 min incubation at RT with the kit reagents, the absorbance at 492 nm was measured with the Synergy HTX multimode reader.

### In Vitro Biological Characterization—MSC Adhesion Assay

MSCs at passage 5 were seeded at a density of 5000 cells per well on substrates. Cells were then incubated for 7 h in serum‐free medium and afterward rinsed with PBS and fixed for immunofluorescent staining (protocol described above). Cytoskeletal actin filaments (F‐actin) were stained with phalloidin‐Alexa Fluor 546 (A22283, Invitrogen, USA) (1:400) in 0.05% Triton‐X for 1 h. Focal adhesions were stained with mouse anti‐vinculin (V9131, Sigma‐Aldrich, USA) (1:100) in BSA 1% for 1 h, followed by Alexa 488 goat antimouse IgG antibody (R37120, Invitrogen, USA) in 0.05% Triton‐X for 1 h. Nuclei were stained with DAPI (1:1000) in PBS‐Glycine for 5 min. Samples were imaged using a fluorescence microscope (Carl Zeiss LSM 800, Germany) and analyzed with Fiji/ImageJ.^[^ ^114^ ^]^

### In Vitro Biological Characterization—MSC Proliferation Assay

C ells at passage 4 were seeded at a density of 10 000 cells per well in serum‐free medium for 7 h. Afterward, medium was replaced with 10% FBS‐supplemented medium and cells were incubated for 3, 7, 10, and 17 days. At each timepoint, the number of cells was quantified with Alamar Blue (Invitrogen Life Technologies, Belgium), by replacing the existing cell medium with 10% Alamar Blue in complete medium v/v and incubating the cells for 2 h at 37 °C. Subsequently, the Alamar Blue medium was collected and fluorescence intensity (*λ*_excitation_ = 560 nm, *λ*_emission_ = 590 nm) was read using a Synergy HTX multimode reader. After each measurement, the remaining Alamar Blue was removed and cells were washed twice with PBS, supplemented with fresh medium and incubated until the next timepoint.

### In Vitro Biological Characterization—MSC Mineralization Assay

MSCs at passage 4 were seeded on the surfaces and incubated for 21 days as described for the proliferation assay. Afterwards, cells were washed twice with PBS and fixed with 4% PFA in PBS for 30 min. After washing the cells with PBS, calcium deposits were stained with 40 mm Alizarin Red S (Sigma‐Aldrich, USA) solution (pH = 7.4) for 20 min at RT while gently shaking. When incubation time was over, samples were washed with distilled water until the unincorporated dye was completely removed. Cells were imaged using an Olympus BX51‐P bright‐field microscope (Olympus Corp., Japan). Quantification of calcium deposits was performed with Fiji/ImageJ software.^[^ ^114^ ^]^

### In Vitro Biological Characterization—MSC ALP Activity

After incubating the cells 14 or 21 days as previously indicated, MSCs were rinsed twice with PBS and lysed with M‐PER. ALP activity was then quantified using the SensoLyte pNPP Alkaline Phosphatase Activity Kit (AnaSpech Inc., USA), following the same procedure as described for C2C12 cells.

### In Vitro Biological Characterization—MSC Gene Expression

Gene Expression of osteogenic markers was evaluated by real‐time reverse transcription polymerase chain reaction (RT‐PCR). 20 000 cells per well at passage 4 were seeded on the functionalized Ti disks in serum‐free medium for 7 h. Subsequently, medium was replaced with fresh basal medium and cells were cultured for 1, 4, or 7 days. After each incubation period, cells were lysed and total RNA was extracted and purified using the RNeasy Mini Kit columns (Qiagen, Germany). RNA quantification was done using a Take3 microvolume plate (Bio‐Tek, USA) through spectrophotometry. RNA was then retrotranscribed to cDNA using the QuantiTect Reverse Transcription kit for RT‐PCR (Qiagen, Germany). RT‐PCR was carried out on a Mic real time PCR cycler (Bio Molecular Systems, Australia) and gene expression was assessed by QuantiFast SYBR Green RT‐PCR Kit (Qiagen, Germany). mRNA expression was normalized to the housekeeping gene Glyceraldehyde 3‐phosphate dehydrogenase (GAPDH) and the relative gene expression levels were evaluated using 2^ΔΔ‐Ct^ method. Primer sequences are listed in Table S1 (Supporting Information).

### In Vivo Experiments—Implants Preparation

Disk‐like Ti implants (5 mm diameter, 2 mm height) with cylindrical holes of 0.5 mm diameter with an equidistance of 0.3 mm were functionalized either with RGD‐DWIVA, cRGD‐DWIVA, or cRGD‐cDWIVA by immersing the implants in the peptide solution at 100 µm. To ensure total functionalization, implants were incubated during 12 h facing up, followed by another 12 h incubation with the samples facing down. Afterward, functionalized implants were sterilized with 70% ethanol v/v for 5 min and allowed to dry in sterile conditions. In total, four different conditions were used, using Ti as a control group and the three different biomimetic peptides (RGD‐DWIVA, cRGD‐DWIVA, and cRGD‐cDWIVA) to evaluate the osteogenic potential of the functionalized implants.

### In Vivo Experiments—In Vivo Implantation

in vivo bone formation was evaluated on a rat calvarial defect. In detail, 12 week‐old healthy male Sprague‐Dawley rats were used. The animal caring, housing and experimental protocols (DKU‐18‐032) were approved by the Animal Care and Use Committee at Dankook University, Republic of Korea. Animals were naturalized for 5–7 days before surgery and each rat was housed in a separate cage under temperature and humidity controlled environment, exposed to a 12 h light‐dark cycle, and had free access to water and food. The animals were randomly assigned to five experimental groups (*n* = 5) including the three study groups (RGD‐DWIVA, cRGD‐DWIVA, and cRGD‐cDWIVA) and two control groups (noncoated Ti and empty). The disks were implanted under general anesthesia using intramuscular injection of a mixture of ketamine (80 mg kg^−1^) and xylazine (10 mg kg^−1^).

Before opening the calvarial skin area, shaving over the cranial lesion was performed and the surgical site was cleaned with iodine and 70% ethanol. Afterward, a linear skin incision was made by a surgical blade (No.10). A full‐thickness flap was peeled, and the calvarial bone was exposed. Then, 5 mm circular bone defects were made in the right and left sides of the parietal bone under cooling conditions with sterile saline using a dental hand‐piece and a 5 mm diameter trephine drill (South Korea). Finally, implantation was done, and the subcutaneous tissues and periosteum were sutured with absorbable sutures (4‐0 Vicryl, Ethicon, Germany), and the skin was closed with nonabsorbable suture material (4‐0 Prolene, Ethicon, Germany). After surgery, the animals were monitored regularly for possible clinical signs of infection, inflammation, and any injurious reaction. The animals were euthanized 14 weeks postsurgery by CO_2_ inhalation, and the Ti disks were harvested together with the surrounding bone and fixed in 10% neutral buffered formalin for at least 24 h at RT.

### In Vivo Experiments—Histological Preparation and Evaluation

For The histological analysis, tissues were fixed and included in a resin block. The fixed samples were stained with hematoxylin and eosin (H&E) and Goldner's trichrome (GT) following standard protocols to assess new bone formation. Samples were imaged using a light microscope (IX71, Olympus, Japan) and analyzed by ImageJ software.

### Statistical Analysis

All data presented in this work are given as mean values ± standard deviation. When normal distribution of the samples was observed for the different conditions, one‐way ANOVA test was applied, followed by a post‐hoc pairwise comparison using Tukey's (for homogeneous variances between conditions) or Tamhanne (for non‐homogeneous variances) test. Otherwise, the nonparametric Kruskal–Wallis test was used. The calculated *p* values were considered significant, if *p* < 0.05. SPSS Statistics 24.0 software (IBM, USA) was used for statistical analysis.

## Conflict of Interest

The authors declare no conflict of interest.

## Author Contributions

LL.O.C., H.M.G., N.M., E.A.C.A., J.H.L., and C.M.M. conceived the experiments. LL.O.C., H.M.G., and N.M. performed the experiments. Y.W.J., E.A.C.A., H.W.K., and J.H.L. provided materials and expertise. E.A.C.A. and M.P.G. provided critique and context for the data. H.W.K., M.P.G., J.H.L., and C.M.M. funded the work. LL.O.C., H.M.G., N.M., and C.M.M. analyzed the data. LL.O.C., H.M.G., N.M., and C.M.M. prepared the figures and wrote the manuscript. All authors read and commented on the manuscript.

## Supporting information

Supporting Information

## Data Availability

The data that support the findings of this study are available from the corresponding author upon reasonable request.

## References

[1] A. K. Shah , J. Lazatin , R. K. Sinha , T. Lennox , N. J. Hickok , R. S. Tuan , Biol. Cell 1999, 91, 131. 10399828 10.1016/s0248-4900(99)80037-9

[2] J. J. Rice , M. M. Martino , L. De Laporte , F. Tortelli , P. S. Briquez , J. A. Hubbell , Adv. Healthcare Mater. 2012, 2, 57. 10.1002/adhm.201200197 23184739

[3] M. M. Martino , P. S. Briquez , K. Maruyama , J. A. Hubbell , Adv. Drug Delivery Rev. 2015, 94, 41. 10.1016/j.addr.2015.04.007 25895621

[4] X. Xu , L. Zheng , Q. Yuan , G. Zhen , J. L. Crane , X. Zhou , X. Cao , Bone Res. 2018, 6, 2. 29423331 10.1038/s41413-017-0005-4 PMC5802812

[5] M. J. Dalby , A. J. García , M. Salmeron‐Sanchez , Nat. Rev. Mater. 2018, 3, 17091.

[6] H. Lin , Y. Tang , T. P. Lozito , N. Oyster , B. Wang , R. S. Tuan , Stem Cell Res. Ther. 2019, 10, 254. 31412905 10.1186/s13287-019-1350-6 PMC6694509

[7] C. E. Vantucci , L. Krishan , A. Cheng , A. Prather , K. Roy , R. E. Guldberg , Biomater. Sci. 2021, 9, 1668. 33409509 10.1039/d0bm01728k PMC8256799

[8] R. Aquino‐Martínez , N. Artigas , B. Gá Mez , J. L. Rosa , F. Ventura , PLoS One 2017, 12, e0178158. 28542453 10.1371/journal.pone.0178158 PMC5444778

[9] A. Alba‐Perez , V. Jayawarna , P. G. Childs , M. J. Dalby , M. Salmeron‐Sanchez , Mater. Sci. Eng. C 2020, 113, 110966. 10.1016/j.msec.2020.110966 32487385

[10] A. Ho‐Shui‐Ling , J. Bolander , L. E. Rustom , A. W. Johnson , F. P. Luyten , C. Picart , Biomaterials 2018, 180, 143. 30036727 10.1016/j.biomaterials.2018.07.017 PMC6710094

[11] F. Gilde , L. Fourel , R. Guillot , I. Pignot‐Paintrand , T. Okada , V. Fitzpatrick , T. Boudou , C. Albiges‐Rizo , C. Picart , Acta Biomater. 2016, 46, 55. 27633320 10.1016/j.actbio.2016.09.014 PMC5113753

[12] J. N. Zara , R. K. Siu , X. Zhang , J. Shen , R. Ngo , M. Lee , W. Li , M. Chiang , J. Chung , J. Kwak , B. M. Wu , K. Ting , C. Soo , Tissue Eng. – Part A 2011, 17, 1389. 21247344 10.1089/ten.tea.2010.0555 PMC3079169

[13] G. Schmidmaier , P. Schwabe , C. Strobel , B. Wildemann , Injury 2008, 39, S37. 10.1016/S0020-1383(08)70014-7 18804572

[14] E. J. Carragee , E. L. Hurwitz , B. K. Weiner , Spine J. 2011, 11, 471. 21729796 10.1016/j.spinee.2011.04.023

[15] A. W. James , G. LaChaud , J. Shen , G. Asatrian , V. Nguyen , X. Zhang , K. Ting , C. Soo , Tissue Eng., Part B 2016, 22, 284. 10.1089/ten.teb.2015.0357 PMC4964756 26857241

[16] A. Civantos , E. Martínez‐Campos , V. Ramos , C. Elvira , A. Gallardo , A. Abarrategi , ACS Biomater. Sci. Eng. 2017, 3, 1245. 33440513 10.1021/acsbiomaterials.6b00604

[17] O. Chaudhuri , L. Gu , D. Klumpers , M. Darnell , S. A. Bencherif , J. C. Weaver , N. Huebsch , H.‐P. Lee , E. Lippens , G. N. Duda , D. J. Mooney , Nat. Mater. 2016, 15, 326. 26618884 10.1038/nmat4489 PMC4767627

[18] W. L. Murphy , T. C. McDevitt , A. J. Engler , Nat. Mater. 2014, 13, 547. 24845994 10.1038/nmat3937 PMC4163547

[19] M. Alipour , M. Baneshi , S. Hosseinkhani , R. Mahmoudi , A. Jabari Arabzadeh , M. Akrami , J. Mehrzad , H. Bardania , Biomed. Mater. Res. A 2020, 108, 839. 10.1002/jbm.a.36862 31854488

[20] S. L. Bellis , Biomaterials 2011, 32, 4205. 21515168 10.1016/j.biomaterials.2011.02.029 PMC3091033

[21] M. Salmerón‐Sánchez , M. J. Dalby , Chem. Commun. 2016, 52, 13327. 10.1039/c6cc06888j 27722261

[22] C. Mas‐Moruno , B. Su , M. J. Dalby , Adv. Healthcare Mater. 2019, 8, 1801103. 10.1002/adhm.201801103 30468010

[23] M. M. Martino , F. Tortelli , M. Mochizuki , S. Traub , D. Ben‐David , G. A. Kuhn , R. Müller , E. Livne , S. A. Eming , J. A. Hubbell , Sci. Transl. Med. 2011, 3, 100ra89. 10.1126/scitranslmed.3002614 21918106

[24] Z. A. Cheng , A. Alba‐Perez , C. Gonzalez‐Garcia , H. Donnelly , V. Llopis‐Hernandez , V. Jayawarna , P. Childs , D. W. Shields , M. Cantini , L. Ruiz‐Cantu , A. Reid , J. F. C. Windmill , E. S. Addison , S. Corr , W. G. Marshall , M. J. Dalby , M. Salmeron‐Sanchez , Adv. Sci. 2019, 6, 1800361. 10.1002/advs.201800361 PMC6343071 30693176

[25] V. Llopis‐Hernández , M. Cantini , C. González‐García , Z. A. Cheng , J. Yang , P. M. Tsimbouri , A. J. García , M. J. Dalby , M. Salmerón‐Sánchez , Sci. Adv. 2016, 2, e1600188. 27574702 10.1126/sciadv.1600188 PMC5001810

[26] M. Kisiel , M. M. Martino , M. Ventura , J. A. Hubbell , J. Hilborn , D. A. Ossipov , Biomaterials 2013, 34, 704. 23103154 10.1016/j.biomaterials.2012.10.015

[27] A. Shekaran , J. R. García , A. Y. Clark , T. E. Kavanaugh , A. S. Lin , R. E. Guldberg , A. J. García , Biomaterials 2014, 35, 5453. 24726536 10.1016/j.biomaterials.2014.03.055 PMC4033404

[28] O. Dobre , M. A. G. Oliva , G. Ciccone , S. Trujillo , A. Rodrigo‐Navarro , D. C. Venters , V. Llopis‐Hernandez , M. Vassalli , C. Gonzalez‐Garcia , M. J. Dalby , M. Salmeron‐Sanchez , Adv. Funct. Mater. 2021, 31, 2010225.

[29] S. Trujillo , C. Gonzalez‐Garcia , P. Rico , A. Reid , J. Windmill , M. J. Dalby , M. Salmeron‐Sanchez , Biomaterials 2020, 252, 120104. 32422492 10.1016/j.biomaterials.2020.120104

[30] R. Visser , G. A. Rico‐Llanos , H. Pulkkinen , J. Becerra , J. Controlled Release 2016, 244, 122. 10.1016/j.jconrel.2016.10.024 27794492

[31] A. E. Rodda , L. Meagher , D. R. Nisbet , J. S. Forsythe , Prog. Polym. Sci. 2014, 39, 1312.

[32] D. Yadin , P. Knaus , T. D. Mueller , Cytokine Growth Factor Rev. 2016, 27, 13. 26690041 10.1016/j.cytogfr.2015.11.005

[33] A. Saito , Y. Suzuki , S. I. Ogata , C. Ohtsuki , M. Tanihara , J. Biomed. Mater. Res., Part A 2005, 72, 77. 10.1002/jbm.a.30208 15543633

[34] W. Zhang , J. Liu , C. Zhang , X. Yu , B. Zhong , Bioorg. Chem. 2021, 116, 105382. 34598087 10.1016/j.bioorg.2021.105382

[35] J. Y. Lee , J. E. Choo , Y. S. Choi , J. S. Suh , S. J. Lee , C. P. Chung , Y. J. Park , Biomaterials 2009, 30, 3532. 19345406 10.1016/j.biomaterials.2009.03.018

[36] L. Oliver‐Cervelló , H. Martin‐Gómez , C. Mas‐Moruno , J. Pept. Sci. 2022, 28, e3335. 34031952 10.1002/psc.3335

[37] N. M. Moore , N. J. Lin , N. D. Gallant , M. L. Becker , Acta Biomater. 2011, 7, 2091. 21272672 10.1016/j.actbio.2011.01.019

[38] I. Bilem , P. Chevallier , L. Plawinski , E. D. Sone , M. C. Durrieu , G. Laroche , Acta Biomater. 2016, 36, 132. 27000551 10.1016/j.actbio.2016.03.032

[39] C. M. Madl , M. Mehta , G. N. Duda , S. C. Heilshorn , D. J. Mooney , Biomacromolecules 2014, 15, 445. 24400664 10.1021/bm401726u PMC3930060

[40] D. Halloran , H. W. Durbano , A. Nohe , J. Dev. Biol. 2020, 8, 19. 32933207 10.3390/jdb8030019 PMC7557435

[41] M. C. Gomez‐Puerto , P. Vasudevan Iyengar , A. García De Vinuesa , P. Ten Dijke , G. Sanchez‐Duffhues , J. Pathol. 2019, 247, 9. 30246251 10.1002/path.5170 PMC6587955

[42] O. F. Zouani , J. Kalisky , E. Ibarboure , M. C. Durrieu , Biomaterials 2013, 34, 2157. 23290467 10.1016/j.biomaterials.2012.12.007

[43] T. Kirsch , EMBO J. 2000, 19, 3314. 10880444 10.1093/emboj/19.13.3314 PMC313944

[44] J. Nickel , M. K. Dreyer , T. Kirsch , W. Sebald , J. Bone Jt. Surg. 2001, 83‐A, S7. 11263668

[45] A. Saito , Y. Suzuki , S.‐I. Ogata , C. Ohtsuki , M. Tanihara , Biochim. Biophys. Acta 2003, 1651, 60. 14499589 10.1016/s1570-9639(03)00235-8

[46] A. Saito , Y. Suzuki , S. I. Ogata , C. Ohtsuki , M. Tanihara , J. Biomed. Mater. Res., Part A 2004, 70, 115. 10.1002/jbm.a.30071 15174115

[47] S. Keller , J. Nickel , J. L. Zhang , W. Sebald , T. D. Mueller , Nat. Struct. Mol. Biol. 2004, 11, 481. 15064755 10.1038/nsmb756

[48] A. Kotzsch , J. Nickel , A. Seher , K. Heinecke , L. Van Geersdaele , T. Herrmann , W. Sebald , T. D. Mueller , J. Biol. Chem. 2008, 283, 5876. 18160401 10.1074/jbc.M706029200

[49] O. Drevelle , A. Daviau , M. A. Lauzon , N. Faucheux , Biomaterials 2013, 34, 1051. 23164422 10.1016/j.biomaterials.2012.10.066

[50] M. Beederman , J. D. Lamplot , G. Nan , J. Wang , X. Liu , L. Yin , R. Li , W. Shui , H. Zhang , S. H. Kim , W. Zhang , J. Zhang , Y. Kong , S. Denduluri , M. R. Rogers , A. Pratt , R. C. Haydon , H. H. Luu , J. Angeles , L. L. Shi , T.‐C. He , J. Biomed. Sci. Eng. 2013, 6, 32. 26819651 10.4236/jbise.2013.68A1004 PMC4725591

[51] A. Yamaguchi , T. Ishizuya , N. Kintou , Y. Wada , T. Katagiri , J. M. Wozney , V. Rosen , S. Yoshiki , Biochem. Biophys. Res. Commun. 1996, 220, 366. 8645311 10.1006/bbrc.1996.0411

[52] T. L. M. Pohl , J. H. Boergermann , G. K. Schwaerzer , P. Knaus , E. A. Cavalcanti‐Adam , Acta Biomater. 2012, 8, 772. 22040684 10.1016/j.actbio.2011.10.019

[53] T. Katagiri , J. Cell Biol. 1994, 127, 1755. 7798324 10.1083/jcb.127.6.1755 PMC2120318

[54] E. H. Schwab , T. L. M. Pohl , T. Haraszti , G. K. Schwaerzer , C. Hiepen , J. P. Spatz , P. Knaus , E. A. Cavalcanti‐Adam , Nano Lett. 2015, 15, 1526. 25668064 10.1021/acs.nanolett.5b00315

[55] G. Chen , H. Xu , Y. Yao , T. Xu , M. Yuan , X. Zhang , Z. Lv , M. Wu , Front. Cell Dev. Biol. 2020, 8, 135. 32211409 10.3389/fcell.2020.00135 PMC7075941

[56] J. Yang , P. Shi , M. Tu , Y. Wang , M. Liu , F. Fan , M. Du , Food Sci. Hum. Wellness 2014, 3, 127.

[57] The UniProt Consortium , Nucl. Acids Res. 2021, 49, D480. 33237286 10.1093/nar/gkaa1100 PMC7778908

[58] X. He , J. Ma , E. Jabbari , Langmuir 2008, 24, 12508. 18837524 10.1021/la802447v

[59] L. Oliver‐Cervelló , H. Martin‐Gómez , L. Reyes , F. Noureddine , E. Ada Cavalcanti‐Adam , M. P. Ginebra , C. Mas‐Moruno , Adv. Healthcare Mater. 2021, 10, 2001757. 10.1002/adhm.202001757 33336559

[60] H. Martin‐Gómez , L. Oliver‐Cervelló , J. Buxadera‐Palomero , M. P. Ginebra , C. Mas‐Moruno , ChemBioChem 2021, 22, 839. 33094896 10.1002/cbic.202000670

[61] C. Mas‐Moruno , R. Fraioli , F. Rechenmacher , S. Neubauer , T. G. Kapp , H. Kessler , Angew. Chem., Int. Ed. 2016, 55, 7048. 10.1002/anie.201509782 27258759

[62] T. G. Kapp , F. Rechenmacher , S. Neubauer , O. V. Maltsev , E. A. Cavalcanti‐Adam , R. Zarka , U. Reuning , J. Notni , H.‐J. Wester , C. Mas‐Moruno , J. Spatz , B. Geiger , H. Kessler , Sci. Rep. 2017, 7, 39805. 28074920 10.1038/srep39805 PMC5225454

[63] R. Haubner , R. Gratias , B. Diefenbach , S. L. Goodman , A. Jonczyk , H. Kessler , J. Am. Chem. Soc. 1996, 118, 7461.

[64] P. Kord Forooshani , B. P. Lee , J. Polym. Sci., Part A: Polym. Chem. 2017, 55, 9. 10.1002/pola.28368 PMC5132118 27917020

[65] M. Pagel , R. Hassert , T. John , K. Braun , M. Wießler , B. Abel , A. G. Beck‐sickinger , Angew. Chem. 2016, 55, 4826. 26938787 10.1002/anie.201511781

[66] H. Lee , N. F. Scherer , P. B. Messersmith , Proc. Natl. Acad. Sci. USA 2006, 103, 12999. 16920796 10.1073/pnas.0605552103 PMC1559742

[67] H. Lee , S. M. Dellatore , W. M. Miller , P. B. Messersmith , Science 2007, 318, 426. 17947576 10.1126/science.1147241 PMC2601629

[68] W. Tang , G. M. Policastro , G. Hua , K. Guo , J. Zhou , C. Wesdemiotis , G. L. Doll , M. L. Becker , J. Am. Chem. Soc. 2014, 136, 16357. 25343707 10.1021/ja508946h

[69] Y. Li , M. Qin , Y. Li , Y. Cao , W. Wang , Langmuir 2014, 30, 4358. 24716607 10.1021/la501189n

[70] M. Hoyos‐Nogués , F. Velasco , M. P. Ginebra , J. M. Manero , F. J. Gil , C. Mas‐Moruno , ACS Appl. Mater. Interfaces 2017, 9, 21618. 28594999 10.1021/acsami.7b03127

[71] M. Pagel , A. G. Beck‐Sickinger , Biol. Chem. 2017, 398, 3. 27636830 10.1515/hsz-2016-0204

[72] G. Pan , S. Sun , W. Zhang , R. Zhao , W. Cui , F. He , L. Huang , S. H. Lee , K. J. Shea , Q. Shi , H. Yang , J. Am. Chem. Soc. 2016, 138, 15078. 27778505 10.1021/jacs.6b09770

[73] M. Hoyos‐Nogués , E. Falgueras‐Batlle , M. P. Ginebra , J. M. Manero , J. Gil , C. Mas‐Moruno , Int. J. Mol. Sci. 2019, 20, 1429. 30901841 10.3390/ijms20061429 PMC6470513

[74] C. Mas‐Moruno , R. Fraioli , F. Albericio , J. M. Manero , F. J. Gil , ACS Appl. Mater. Interfaces 2014, 6, 6525. 24673628 10.1021/am5001213

[75] R. Fraioli , K. Dashnyam , J. H. Kim , R. A. Perez , H. W. Kim , J. Gil , M. P. Ginebra , J. M. Manero , C. Mas‐Moruno , Acta Biomater. 2016, 43, 269. 27481289 10.1016/j.actbio.2016.07.049

[76] S. J. Xiao , M. T. Ext Or , N. D. Spe Nce R , M. L. A. Wie Nd , B. K. Ller , H. Sigrist , J. Mater. Sci. Mater. Med. 1997, 8, 867. 15348806 10.1023/a:1018501804943

[77] R. Fraioli , F. Rechenmacher , S. Neubauer , J. M. Manero , J. Gil , H. Kessler , C. Mas‐Moruno , Colloids Surf., B 2015, 128, 191. 10.1016/j.colsurfb.2014.12.057 25637448

[78] R. Fraioli , S. Neubauer , F. Rechenmacher , B. M. Bosch , K. Dashnyam , J. H. Kim , R. A. Perez , H. W. Kim , F. J. Gil , M. P. Ginebra , J. M. Manero , H. Kessler , C. Mas‐Moruno , Biomater. Sci. 2019, 7, 1281. 30735211 10.1039/c8bm01466c

[79] S. P. Massia , J. A. Hubbell , J. Cell Biol. 1991, 114, 1089. 1714913 10.1083/jcb.114.5.1089 PMC2289117

[80] J. Sefkow‐Werner , P. Machillot , A. Sales , E. Castro‐Ramirez , M. Degardin , D. Boturyn , E. A. Cavalcanti‐Adam , C. Albiges‐Rizo , C. Picart , E. Migliorini , Acta Biomater. 2020, 114, 90. 32673751 10.1016/j.actbio.2020.07.015

[81] P. W. Kämmerer , M. Helle , J. Brieger , M. O. Klein , B. Al‐Nawas , M. Gabriel , Eur. Cells Mater. 2011, 21, 364. 10.22203/ecm.v021a27 21484706

[82] M. Heller , V. V. Kumar , A. Pabst , J. Brieger , B. Al‐Nawas , P. W. Kämmerer , J. Biomed. Mater. Res. Part A 2018, 106, 419. 10.1002/jbm.a.36255 28971567

[83] R. Fraioli , P. M. Tsimbouri , L. E. Fisher , A. H. Nobbs , B. Su , S. Neubauer , F. Rechenmacher , H. Kessler , M.‐P. Ginebra , M. J. Dalby , J. M. Manero , C. Mas‐Moruno , Sci. Rep. 2017, 7, 16363. 29180787 10.1038/s41598-017-16385-3 PMC5703844

[84] M. J. P. Biggs , R. G. Richards , S. McFarlane , C. D. W. Wilkinson , R. O. C. Oreffo , M. J. Dalby , J. R. Soc. Interface 2008, 5, 1231. 18348958 10.1098/rsif.2008.0035 PMC3226994

[85] M. J. P. Biggs , R. G. Richards , N. Gadegaard , C. D. W. Wilkinson , R. O. C. Oreffo , M. J. Dalby , Biomaterials 2009, 30, 5094. 19539986 10.1016/j.biomaterials.2009.05.049

[86] H. Cai , J. Zou , W. Wang , A. Yang , Mol. Med. Rep. 2021, 23, 125. 33300084 10.3892/mmr.2020.11764 PMC7751477

[87] M. Zaidi , Nat. Med. 2007, 13, 791. 17618270 10.1038/nm1593

[88] S. Gromolak , A. Krawczenko , A. Antończyk , K. Buczak , Z. Kiełbowicz , A. Klimczak , Int. J. Mol. Sci. 2020, 21, 9726. 33419255 10.3390/ijms21249726 PMC7766718

[89] M. B. Meyer , N. A. Benkusky , J. W. Pike , J. Biol. Chem. 2014, 289, 16016. 24764292 10.1074/jbc.M114.552216 PMC4047377

[90] H. Sepulveda , R. Aguilar , C. P. Prieto , F. Bustos , S. Aedo , J. Lattus , B. van Zundert , V. Palma , M. Montecino , J. Cell. Physiol. 2017, 232, 2519. 27689934 10.1002/jcp.25627

[91] R. Marom , I. Shur , R. Solomon , D. Benayahu , J. Cell. Physiol. 2005, 202, 41. 15389528 10.1002/jcp.20109

[92] Q. Wei , T. L. M. Pohl , A. Seckinger , J. P. Spatz , E. A. Cavalcanti‐Adam , Beilstein J. Org. Chem. 2015, 11, 773. 26124879 10.3762/bjoc.11.87 PMC4464188

[93] A. Nohe , S. Hassel , M. Ehrlich , F. Neubauer , W. Sebald , Y. I. Henis , P. Knaus , J. Biol. Chem. 2002, 277, 5330. 11714695 10.1074/jbc.M102750200

[94] R. Sano , A. Nakajima , T. Kawato , M. Maeno , N. Shimizu , J. Hard Tissue Biol. 2017, 26, 177.

[95] K. M. Sinha , X. Zhou , J. Cell. Biochem. 2013, 114, 975. 23225263 10.1002/jcb.24439 PMC3725781

[96] O. Frank , M. Heim , M. Jakob , A. Barbero , D. Schäfer , I. Bendik , W. Dick , M. Heberer , I. Martin , J. Cell. Biochem. 2002, 85, 737. 11968014 10.1002/jcb.10174

[97] C. Granéli , A. Thorfve , U. Ruetschi , H. Brisby , P. Thomsen , A. Lindahl , C. Karlsson , Stem Cell Res. 2014, 12, 153. 24239963 10.1016/j.scr.2013.09.009

[98] F. Viti , M. Landini , A. Mezzelani , L. Petecchia , L. Milanesi , S. Scaglione , PLoS One 2016, 11, e0148173. 26828589 10.1371/journal.pone.0148173 PMC4734718

[99] C. Shen , C. Yang , S. Xu , H. Zhao , Cell Biosci. 2019, 9, 17. 30792848 10.1186/s13578-019-0281-3 PMC6371545

[100] M. J. P. Biggs , M. J. Dalby , Proc. Inst. Mech. Eng. Part H J. Eng. Med. 2010, 224, 1441. 10.1243/09544119JEIM775 PMC3058380 21287830

[101] L. Yang , L. Ge , P. van Rijn , ACS Appl. Mater. Interfaces 2020, 12, 25591. 32423202 10.1021/acsami.0c05012 PMC7291345

[102] K. A. Kilian , B. Bugarija , B. T. Lahn , M. Mrksich , Proc. Natl. Acad. Sci. USA 2010, 107, 4872. 20194780 10.1073/pnas.0903269107 PMC2841932

[103] R. McBeath , D. M. Pirone , C. M. Nelson , K. Bhadriraju , C. S. Chen , Dev. Cell 2004, 6, 483. 15068789 10.1016/s1534-5807(04)00075-9

[104] M. Zhu , K. Zhang , L. Feng , S. Lin , Q. Pan , L. Bian , G. Li , Bioact. Mater. 2021, 6, 1353. 33210028 10.1016/j.bioactmat.2020.11.002 PMC7658495

[105] X. Chen , N. C.‐H. Lai , K. Wei , R. Li , M. Cui , B. Yang , S. H. D. Wong , Y. Deng , J. Li , X. Shuai , L. Bian , ACS Nano 2020, 14, 4027. 32223215 10.1021/acsnano.9b08564

[106] B. Elmengaard , J. E. Bechtold , K. Søballe , Biomaterials 2005, 26, 3521. 15621242 10.1016/j.biomaterials.2004.09.039

[107] B. Elmengaard , J. E. Bechtold , K. Søballe , J. Biomed. Mater. Res. Part A 2005, 75A, 249. 10.1002/jbm.a.30301 16106438

[108] H. C. Kroese‐Deutman , J. Van Den Dolder , P. H. M. Spauwen , J. A. Jansen , Tissue Eng. 2005, 11, 1867. 16411833 10.1089/ten.2005.11.1867

[109] X. Chen , Y. Gao , Y. Wang , G. Pan , Smart Mater. Med. 2021, 2, 26.

[110] Q. Wei , A. Holle , J. Li , F. Posa , F. Biagioni , O. Croci , A. S. Benk , J. Young , F. Noureddine , J. Deng , M. Zhang , G. J. Inman , J. P. Spatz , S. Campaner , E. A. Cavalcanti‐Adam , Adv. Sci. 2020, 7, 1902931. 10.1002/advs.201902931 PMC7404154 32775147

[111] J. Bai , H. Wang , H. Chen , G. Ge , M. Wang , A. Gao , L. Tong , Y. Xu , H. Yang , G. Pan , P. K. Chu , D. Geng , Biomaterials 2020, 255, 120197. 32563944 10.1016/j.biomaterials.2020.120197

[112] T. Wang , J. Bai , M. Lu , C. Huang , D. Geng , G. Chen , L. Wang , J. Qi , W. Cui , L. Deng , Nat. Commun. 2022, 13, 160. 35013289 10.1038/s41467-021-27816-1 PMC8748715

[113] H. Zhao , Y. Huang , W. Zhang , Q. Guo , W. Cui , Z. Sun , D. Eglin , L. Liu , G. Pan , Q. Shi , ACS Biomater. Sci. Eng. 2018, 4, 2505. 33435114 10.1021/acsbiomaterials.8b00261

[114] J. Schindelin , I. Arganda‐Carreras , E. Frise , V. Kaynig , M. Longair , T. Pietzsch , S. Preibisch , C. Rueden , S. Saalfeld , B. Schmid , J. Y. Tinevez , D. J. White , V. Hartenstein , K. Eliceiri , P. Tomancak , A. Cardona , Nat. Methods 2012, 9, 676. 22743772 10.1038/nmeth.2019 PMC3855844

