# Supplementary material for: Mimicking Bone Extracellular Matrix: From BMP‐2‐Derived Sequences to Osteogenic‐Multifunctional Coatings
Source: Adv Healthc Mater. 2022 Aug 15;11(20):2201339. doi: 10.1002/adhm.202201339 (PMC11468143; doi:10.1002/adhm.202201339)
Supplement: Supplementary file 1 — Supporting Information [file ADHM-11-2201339-s001.pdf]

ADVANCED HEALTHCARE MATERIALS

Supporting Information

for *Adv. Healthcare Mater.*, DOI 10.1002/adhm.202201339

Mimicking Bone Extracellular Matrix: From BMP-2-Derived Sequences to Osteogenic-Multifunctional Coatings

*Lluís Oliver-Cervelló, Helena Martin-Gómez, Nandin Mandakhbayar, Young-Woo Jo, Elisabetta Ada Cavalcanti-Adam, Hae-Won Kim, Maria-Pau Ginebra, Jung-Hwan Lee and Carlos Mas-Moruno**

Supporting Information

Mimicking Bone Extracellular Matrix: from BMP-2-derived Sequences to Osteogenic-Multifunctional Coatings

*Lluís Oliver-Cervelló, Helena Martin-Gómez, Nandin Mandakhbayar, Young-Woo Jo, Elisabetta Ada Cavalcanti-Adam, Hae-Won Kim, Maria-Pau Ginebra, Jung-Hwan Lee and Carlos Mas-Moruno**

1. Supplementary results

Table S1. List of primer sequences used in RT-qPCR.

Gene	Type	Primer (5' → 3')
GAPDH	Forward	TTGCCATCAATGACCCCTTCA
	Reverse	CGCCCCACTTGATTTTGGA
Runx2	Forward	AAATGCCTCCGCTGTTATGAA
	Reverse	GCTCCGGCCCACAAATCT
Col1A1	Forward	AGGTCCCCCTGGAAAGAA
	Reverse	AATCCTCGAGCACCCTGA
ALP	Forward	ATCTTTGGTCTGGCTCCCATG
	Reverse	TTTCCCGTTCACCGTCCAC
Osterix	Forward	TGCTTGAGGAGGAAGTTCAC
	Reverse	AGGTCACTGCCCACAGAGTA
OPN	Forward	AGCTGGATGACCAGAGTGCT
	Reverse	TGAAATTCATGGCTGTGGAA

Chemical structure of the linear peptides

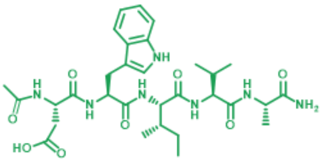

DWIVA

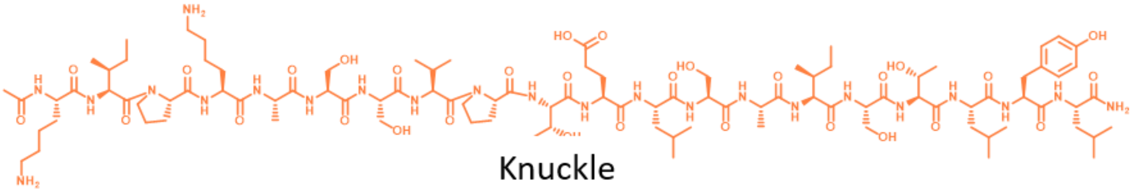

Knuckle

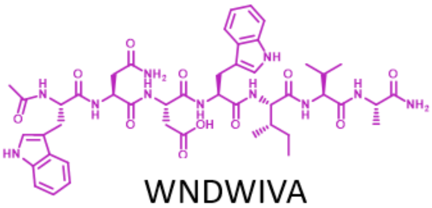

WNDWIVA

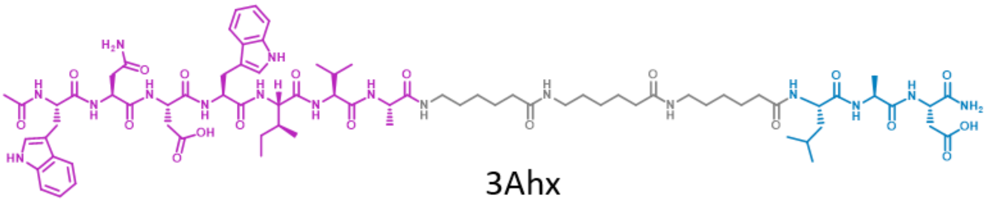

3Ahx

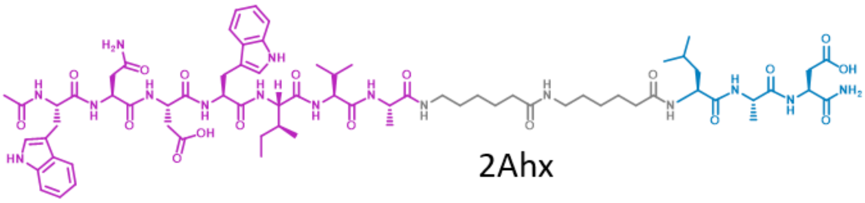

2Ahx

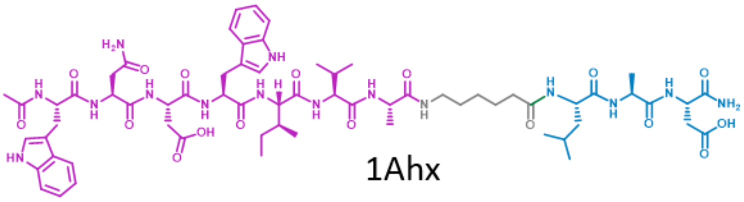

1Ahx

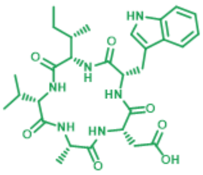

cDWIVA

Figure S1. Chemical structure of the BMP-2-derived peptides covering BMPRs-binding domains. The osteodifferentiation capacity of such peptides was evaluated by studying the power of the peptidic sequences to inhibit myotube formation of C2C12 cells.

Physicochemical characterization of the functionalized Ti substrates

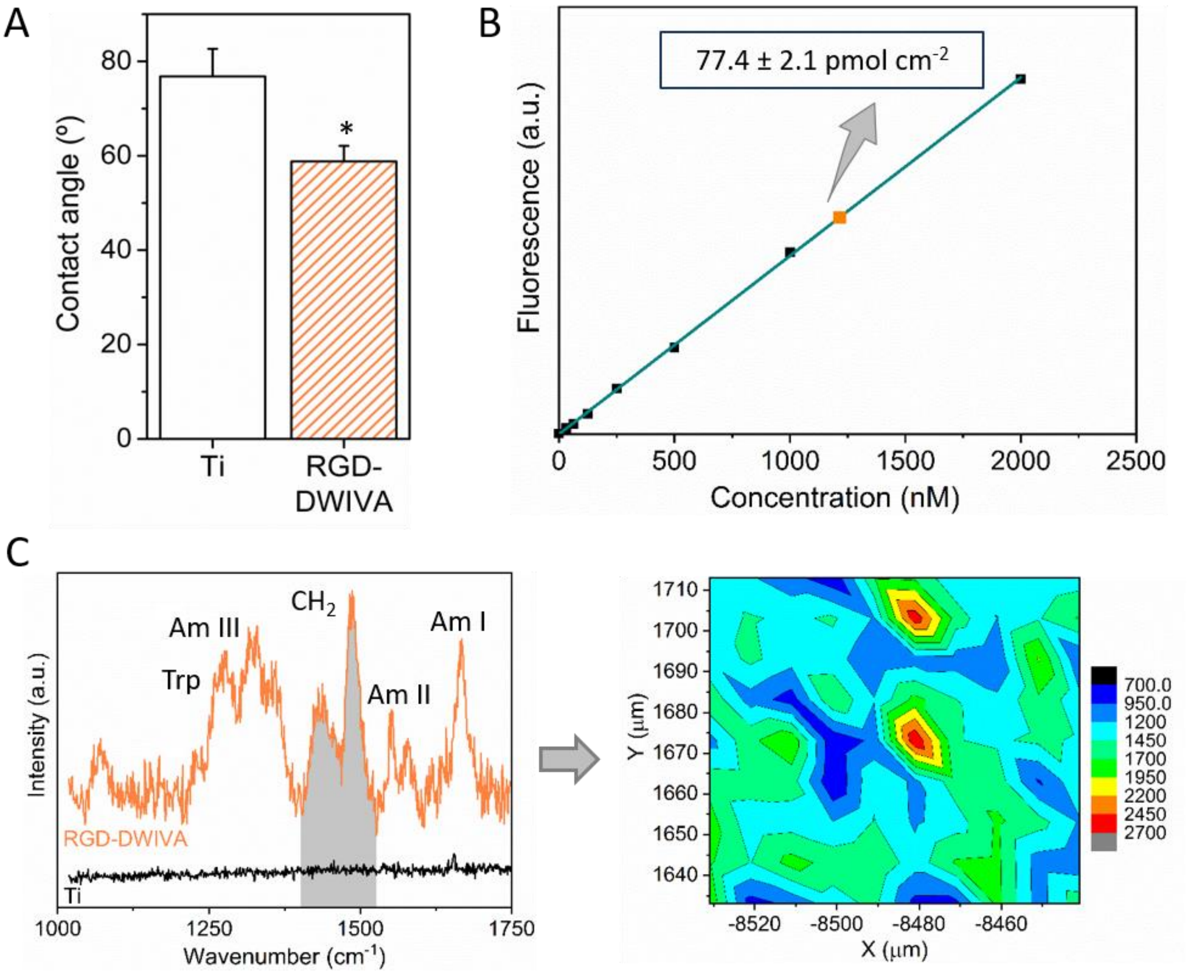

Figure S2. Physicochemical characterization of functionalized Ti with the RGD-DWIVA biomimetic peptide. A) Static contact angle (°) measurements. * indicates statistically significant differences respect Ti ($p < 0.05$). B) The amount of peptide attached to the Ti substrate was quantified using the F-RGD-DWIVA peptide as fluorescent probe (chemical structure and details can be found on [1]). Standard curve used for the quantification. A concentration of 1215.2 nM was obtained, which corresponds to a peptide density of 77.4 ± 2.1 pmol cm^{-2}. C) Raman spectrum of bare Ti and the functionalized Ti with the RGD-DWIVA biomimetic peptide. In left, characteristic peaks corresponding to amides and $-CH_2-$ groups present in the peptide were identified in the Raman spectrum. In right, a mapping of the Raman peak intensity located at 1400-1530 cm^{-1} on the functionalized Ti, on a 90 x 80 µm region. No aggregates were observed all along the functionalized substrate.

Cell adhesion of hMSCs on glass substrates

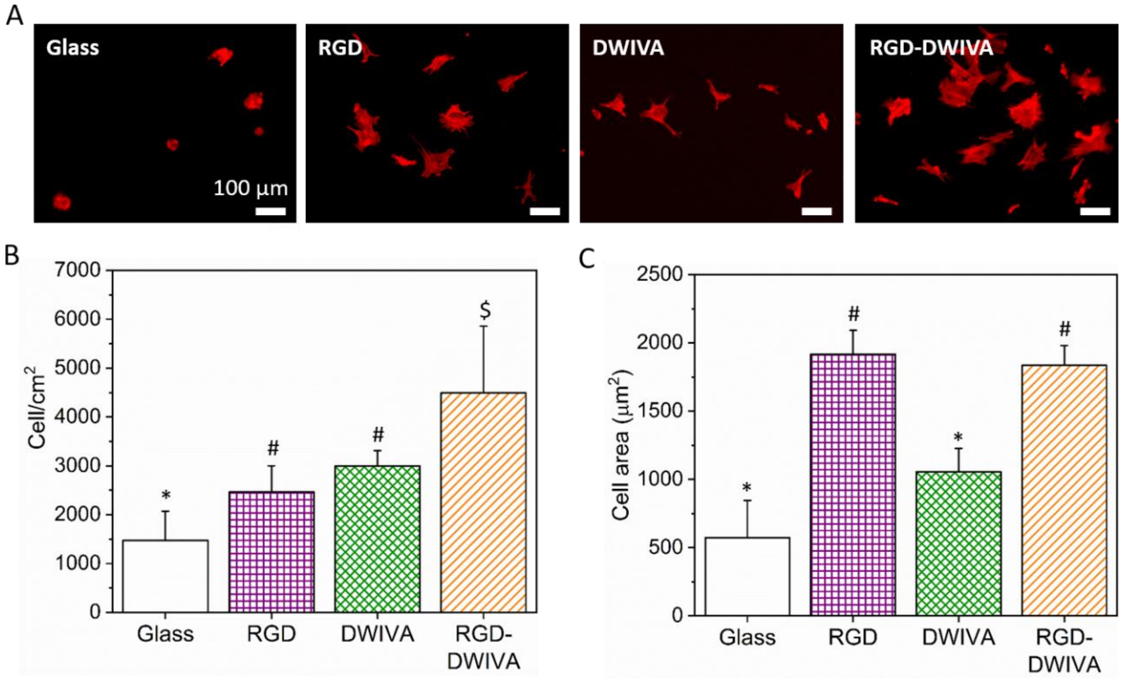

Figure S3. hMSCs adhesion on glass substrates after 7 h incubation in serum-free medium. A) F-actin immunostaining (scale bar = 100 µm), B) quantification of cell numbers and C) projected area. Distinct symbols denote statistically significant differences ($p < 0.05$) between groups. A clear improvement in MSC adhesion was observed on RGD-DWIVA functionalized samples in comparison to untreated glass controls.

Cell proliferation of hMSCs on Ti substrates

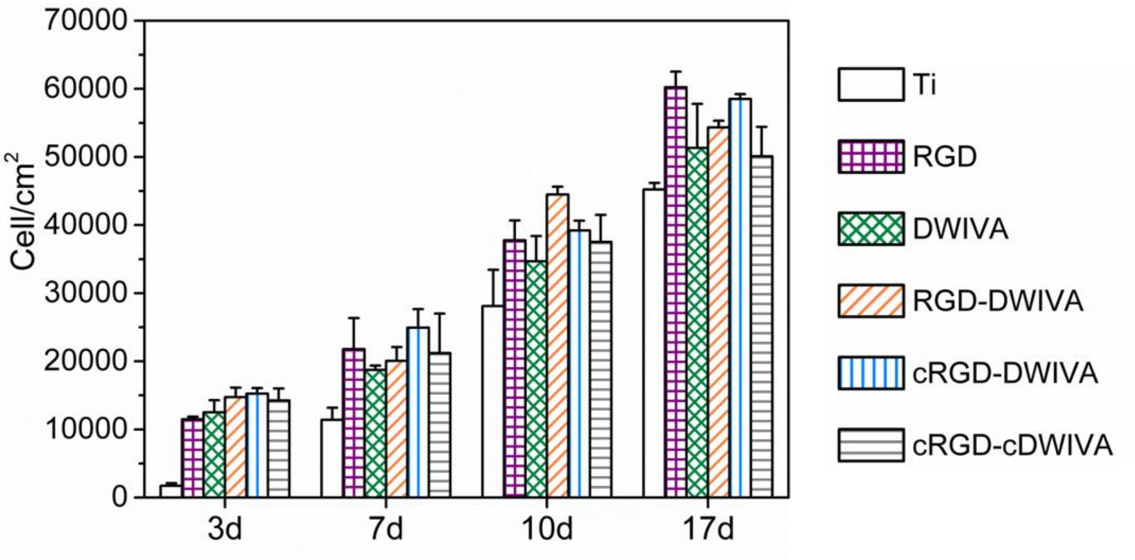

Figure S4. Cell proliferation after 3, 7, 10 and 17 days in culture. Non-functionalized Ti substrates presented the lowest rates of cell proliferation. Interestingly, the synergy observed in the increase of number of adhered cells after 7 h in culture due to the combination of the RGD and DWIVA sequences in the biomimetic peptides is not translated in cell proliferation.

Fibrous tissue and inflammatory cells on Ti implants

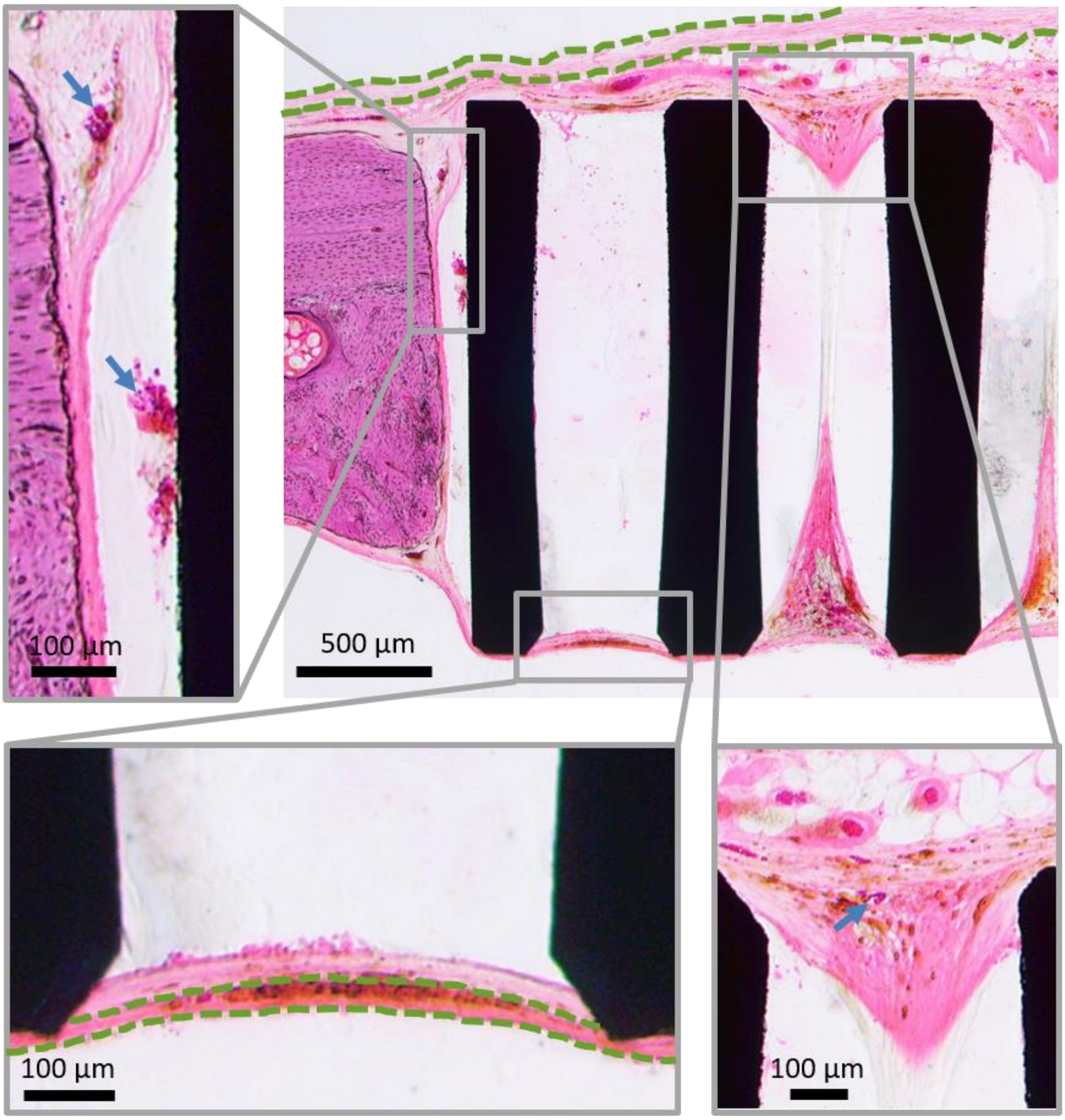

Figure S5. Hematoxylin and eosin staining showing examples of fibrous tissue (green dashed lines) and inflammatory cells (blue arrows) on Ti implant after 14 weeks of implantation. Scale bars are indicated in each image. Fibrous structure thickness was lower than 0.1 mm in all the conditions, and the biomimetic peptides reduced the thickness of such fibrous structure, especially when the cRGD was present on the biomimetic peptides. Moreover, very few inflammatory cells were observed near the Ti implant in all the conditions.

New bone formation on the surrounding of the functionalized implants

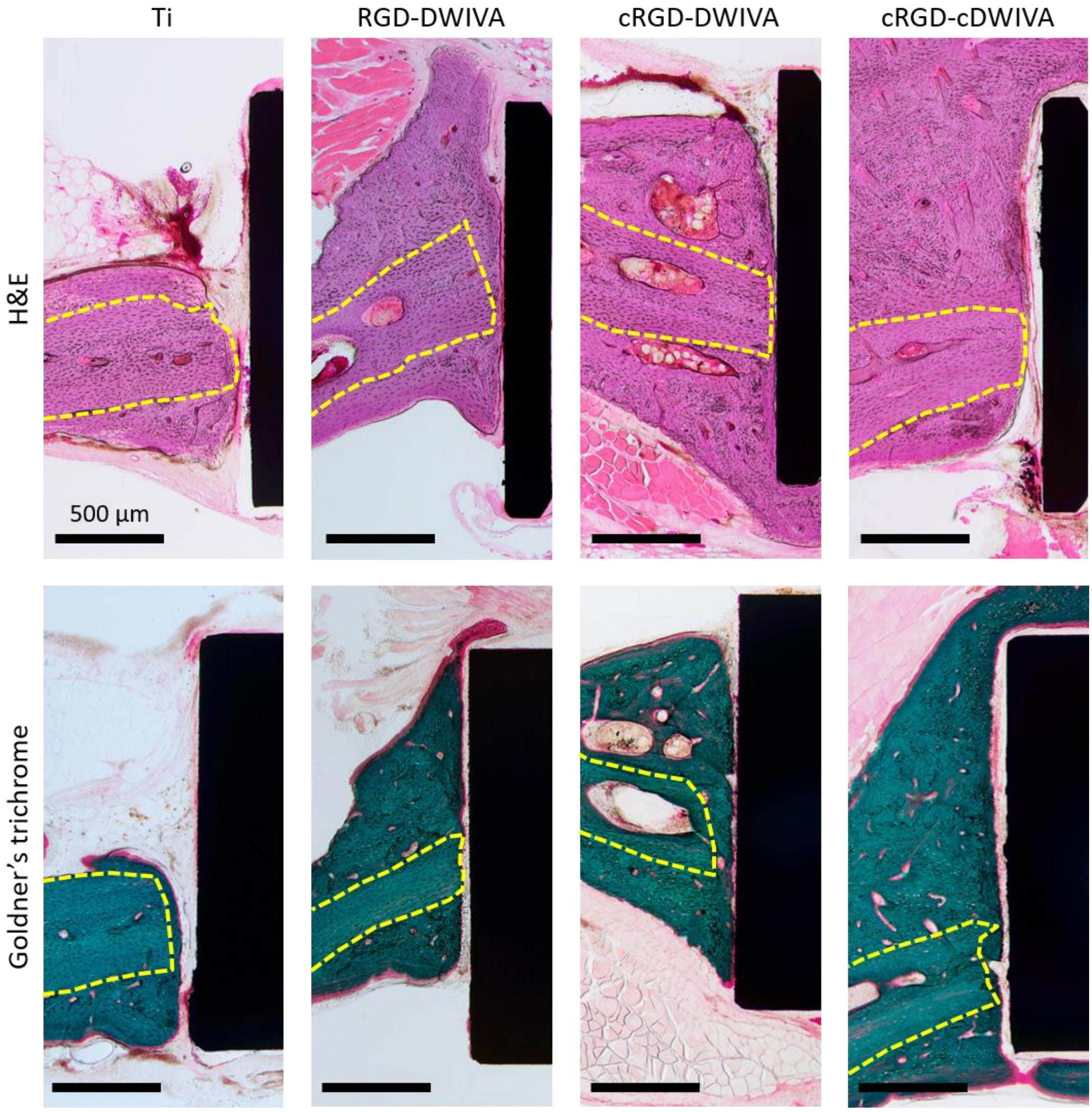

Figure S6. Hematoxylin and eosin (H&E) staining (top) and Goldner's trichrome staining (bottom) showing new bone formation in the surrounding of the implants. Yellow dashed lines denote the area of old bone. Scale bar = 500 µm. Much more quantity of new bone is observed on functionalized implants in comparison to bare Ti.

2. References

[1] L. Oliver-Cervelló, H. Martin-Gómez, L. Reyes, F. Noureddine, E. Ada Cavalcanti-Adam, M. P. Ginebra, C. Mas-Moruno, *Adv. Healthc. Mater.* **2021**, *10*, 2001757.
